# Effect of an eight-week high-intensity interval training programme on circulating sphingolipid levels in middle-aged adults at elevated cardiometabolic risk (SphingoFIT)—Protocol for a randomised controlled exercise trial

**DOI:** 10.1371/journal.pone.0302477

**Published:** 2024-05-08

**Authors:** Justin Carrard, Manuel Hofer, Luisa Prechtl, Eva Fleischlin, Manuel Huber, Hector Gallart-Ayala, Tony Teav, Denis Infanger, Christoph Höchsmann, Karsten Koehler, Timo Hinrichs, Henner Hanssen, Julijana Ivanisevic, Arno Schmidt-Trucksäss

**Affiliations:** 1 Division of Sport and Exercise Medicine, Department of Sport, Exercise and Health, University of Basel, Basel, Switzerland; 2 SportAdo Centre, Children and Adolescent Surgery, Woman-Mother-Child Department, Lausanne University Hospital, Lausanne, Switzerland; 3 Metabolomics Platform, Faculty of Biology and Medicine, University of Lausanne, Lausanne, Switzerland; 4 Department of Health and Sport Sciences, TUM School of Medicine and Health, Technical University of Munich, Munich, Germany; PLOS: Public Library of Science, UNITED KINGDOM

## Abstract

**Introduction:**

Evidence indicates that sphingolipid accumulation drives complex molecular alterations promoting cardiometabolic diseases. Clinically, it was shown that sphingolipids predict cardiometabolic risk independently of and beyond traditional biomarkers such as low-density lipoprotein cholesterol. To date, little is known about therapeutic modalities to lower sphingolipid levels. Exercise, a powerful means to prevent and treat cardiometabolic diseases, is a promising modality to mitigate sphingolipid levels in a cost-effective, safe, and patient-empowering manner.

**Methods:**

This randomised controlled trial will explore whether and to what extent an 8-week fitness-enhancing training programme can lower serum sphingolipid levels of middle-aged adults at elevated cardiometabolic risk (n = 98, 50% females). The exercise intervention will consist of supervised high-intensity interval training (three sessions weekly), while the control group will receive physical activity counselling based on current guidelines. Blood will be sampled early in the morning in a fasted state before and after the 8-week programme. Participants will be provided with individualised, pre-packaged meals for the two days preceding blood sampling to minimise potential confounding. An ’omic-scale sphingolipid profiling, using high-coverage reversed-phase liquid chromatography coupled to tandem mass spectrometry, will be applied to capture the circulating sphingolipidome. Maximal cardiopulmonary exercise tests will be performed before and after the 8-week programme to assess patient fitness changes. Cholesterol, triglycerides, glycated haemoglobin, the homeostatic model assessment for insulin resistance, static retinal vessel analysis, flow-mediated dilatation, and strain analysis of the heart cavities will also be assessed pre- and post-intervention. This study shall inform whether and to what extent exercise can be used as an evidence-based treatment to lower circulating sphingolipid levels.

**Trial registration:**

The trial was registered on www.clinicaltrials.gov (NCT06024291) on August 28, 2023.

## Introduction

Cardiovascular diseases (CVD) are the leading cause of death worldwide and represent a major socioeconomic concern for healthcare systems [1–3]. Early detection and treatment of patients at risk of CVD are crucial to combat this burden effectively [4]. However, clinical evaluations do not easily capture the risk of developing CVD, particularly in patients at intermediate risk [5]. For instance, low-density lipoprotein (LDL) cholesterol, a pillar of cardiovascular risk assessment, was shown to be elevated in only half of the patients hospitalised with coronary artery disease (CAD) [6]. Improving the risk stratification of intermediate-risk patients would enable more individualised primary prevention strategies and eventually reduce the burden related to CVD [5]. In the -omics era, there is a real need for novel pathophysiology-based phenotyping tools to improve cardiovascular risk stratification [7].

### How sphingolipids drive cardiometabolic diseases

**Fig 1:**
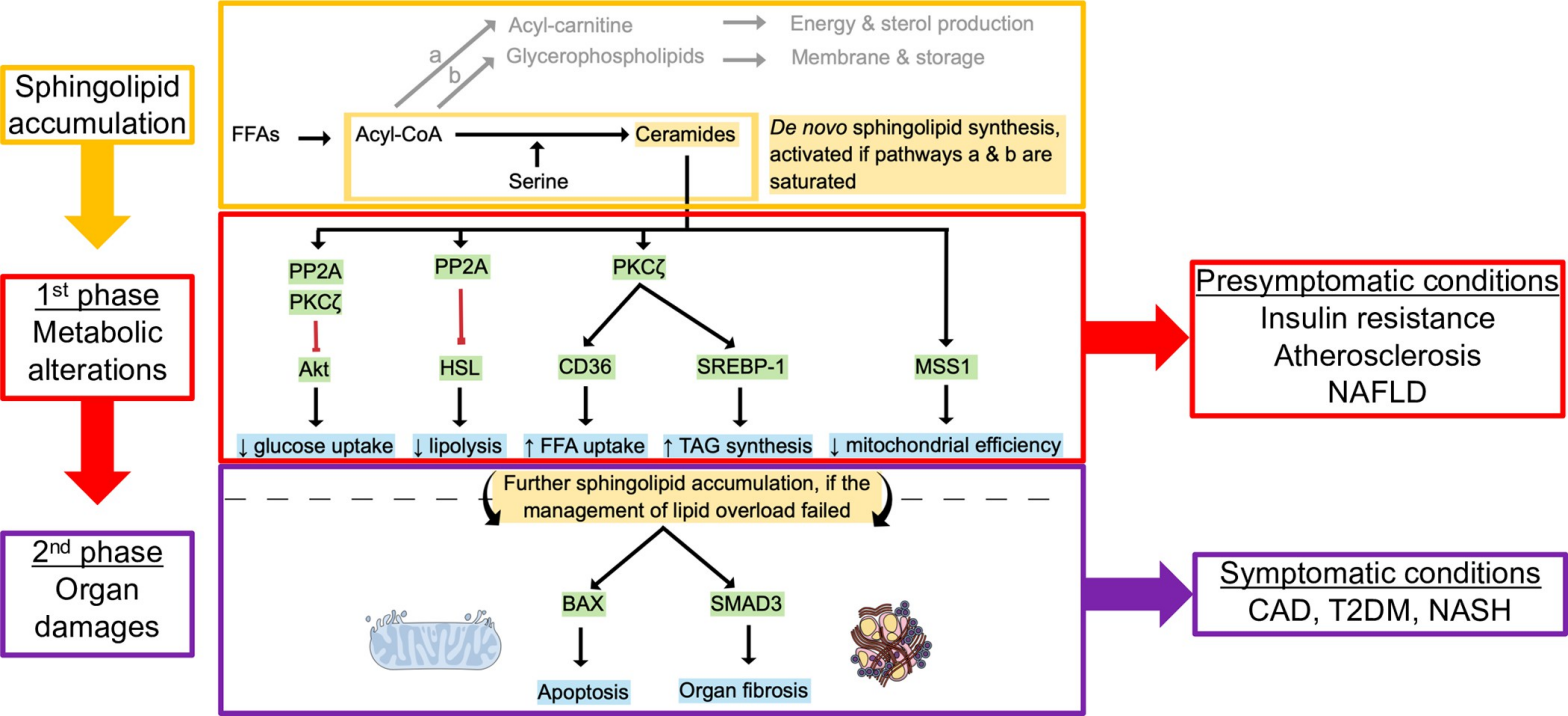
Simplified overview of the molecular alterations and corresponding conditions related to sphingolipid accumulation. Abbreviations: FFA = free fatty acids, acyl-CoA = acyl-coenzyme A, PP2A = protein phosphatase 2 A, PKCζ = protein kinase C zeta, Akt = serine–threonine kinase Akt, HSL = hormone-sensitive lipase, CD36 = cluster of differentiation 36, also known as a fatty acid translocase, SREBP-1 = sterol regulatory element-binding transcription factor 1, TAG = triacylglycerides, MSS1 = mitochondrial fission factor 1, BAX = bcl-2-like protein 4, SMAD3 = Mothers against decapentaplegic homolog 3, NAFLD = non-alcoholic fatty liver disease, T2DM = type 2 diabetes mellitus, NASH = non-alcoholic steatohepatitis, CAD = coronary artery disease. Blue boxes highlight pathophysiological changes and related clinical conditions. This figure is based on [12, 17].

Sphingolipids constitute a family of bioactive lipids that modulate numerous biological processes and are involved in the pathogenesis of CAD, type 2 diabetes mellitus (T2DM), and non-alcoholic fatty liver disease (NAFLD) [8–10]. Sphingolipid-mediated alterations that drive cardiometabolic conditions are illustrated in Fig 1. Briefly, once the triglyceride stores are saturated, in case of overnutrition, reduced energy expenditure or chronic inflammation, lipids in excess are redirected to form sphingolipids [11]. Primarily in muscle and liver cells, sphingolipids accumulate as ceramides, a particular subclass of sphingolipids [10]. Initially, this pathological ceramide accumulation 1) reduces the translocation of glucose transporters to the cell membrane, 2) improves fatty acid storage and uptake, and 3) impairs mitochondrial efficiency, resulting in the production of reactive oxygen species [12]. This initial phase of metabolism alteration leads to peripheral insulin resistance and NAFLD [11, 12]. Failure to manage this sphingolipid overload results in lipotoxicity, a phenomenon characterised by apoptosis and fibrosis, which leads to CAD, non-alcoholic steatohepatitis (NASH), or T2DM [11, 12]. Lastly, ceramides in excess are also carried on LDL, where they drive LDL transcytosis through the endothelium and uptake into macrophages [13, 14]. This results in foam cell formation, vascular inflammation, and atherosclerosis [15]. Situated at the crossroads of overnutrition, dyslipidaemia, and inflammation, sphingolipid metabolism offers a unique opportunity to improve cardiometabolic risk stratification [9, 16].

### Sphingolipid profiling–a pathophysiology-based tool for cardiometabolic risk stratification

The profiling of circulating sphingolipids emerged as a powerful tool for assessing the risk of developing cardiometabolic diseases [12]. Indeed, they predict the occurrence of CVD and T2DM beyond and independently of current prediction tools [18, 19] and can be non-invasively and reliably detected in blood [12]. A machine-learning approach revealed that comprehensive profiling of serum sphingolipids identified patients with CAD independently and more effectively than LDL cholesterol and triglycerides [8]. A score combining the blood level of the four most studied sphingolipid species, i.e., ceramide 16:0, ceramide 18:0, ceramide 24:0, and ceramide 24:1, was developed, and it outperformed the 2019 SCORE of the European Society of Cardiology in terms of cardiovascular risk prediction in primary prevention [20–22]. The same score predicted CAD and stroke mortality beyond conventional lipids in primary and secondary prevention [18, 23]. Precisely ceramides could also predict T2DM occurrence ten years before the disease was diagnosed [19]. This ceramide score has been implemented clinically in private and public hospitals in Finland and at the Cleveland and Mayo Clinic [24, 25]. While the utility of sphingolipid phenotyping in cardiometabolic risk stratification is now well-established [15, 16], little is known about the possibilities of lowering sphingolipid levels.

### Can exercise reduce circulating sphingolipid levels?

To measure circulating sphingolipids in clinical practice, it is essential to provide patients with evidence-based interventions that reduce sphingolipid levels and quantify the reduction expected from such an intervention. Exercise interventions are ideal candidates for mitigating sphingolipid levels. Indeed, exercise is a powerful polypill to prevent and treat cardiometabolic diseases [26]. Exercise interventions are cost-effective, safe, and patient-empowering [27, 28]. The molecular pathways underlying the beneficial effects of exercise are increasingly understood [29]; however, many mechanisms remain to be elucidated [29, 30]. Changes in sphingolipid metabolism might be one of the mechanisms through which exercise optimises cardiometabolic health. Indeed, regular exercise stimulates fatty acid β-oxidation, which redirects lipids toward energy production purposes (pathway a in Fig 1) and could reduce sphingolipid biosynthesis flux [31, 32]. In that way, exercise training could reverse pathological sphingolipid accumulation. This hypothesis is supported by the fact that circulating sphingolipids have been reported to be negatively associated with cardiorespiratory fitness (CRF), a potent health marker [33, 34]. A preliminary study suggested regular exercise could mitigate sphingolipid levels [35]. However, this study was not powered and examined the sphingolipid response to a 12-week moderate-intensity continuous training. Yet it has been demonstrated that high-intensity interval training (HIIT) is a safe [36, 37] and a more effective way to improve CRF [38–40] and insulin sensitivity [41–44] both in healthy individuals and patients with cardiometabolic diseases. Furthermore, this study investigated only a limited number of sphingolipid species (n = 7). In contrast, high-throughput targeted lipidomics methods now allow for the comprehensive analysis of lipid metabolism at the molecular species level [45–47].

## Material and methods

### Aim, objective, and hypothesis

The SphingoFIT study will explore whether and to what extent a supervised CRF-enhancing HIIT-based training programme can lower circulating sphingolipid levels in middle-aged individuals (50% females) at elevated cardiometabolic risk. Accordingly, the objective is to assess the effect of an 8-week CRF-enhancing HIIT-based training programme on a comprehensive panel of plasma sphingolipid species in sedentary adults aged 40 to 60 years (50% females) with overweight or obesity grade 1 but without any other symptomatic cardiometabolic diseases. The hypothesis is that sphingolipid levels will be reduced following the 8-week HIIT-based training programme.

### Endpoints

The primary endpoints will be changes from pre- to post-intervention levels in the four sphingolipid species included in the ceramide-based score (i.e., ceramide 16:0, ceramide 18:0, ceramide 24:0, and ceramide 24:1) [24, 25]. The secondary endpoints will be changes in the other sphingolipids to be targeted (n = 57) in the Homeostatic Model Assessment for Insulin Resistance (HOMA-IR), peak oxygen uptake (VO2peak), flow-mediated dilatation (FMD), retinal vessel analysis, and strain analysis of the heart cavities.

### Study design and general considerations

This prospective two-arm, monocentric, randomised controlled trial will be conducted at the Department of Sport, Exercise, and Health of the University of Basel, Switzerland. It will follow the Declaration of Helsinki and the guidelines of Good Clinical Practice of the World Medical Association in 2013. The Ethics Committee of Northwest and Central Switzerland approved this protocol (EKNZ 2023–01345). Substantial changes to the protocol will be submitted to the Ethics Committee for approval before implementation, as required by Swiss law. The trial was registered on www.clinicaltrials.gov (NCT06024291) on August 28, 2023, and follows the Standard Protocol Items: Recommendations for Interventional Trials (SPIRIT) reporting guidelines [48]. According to the SPIRIT reporting guidelines, all World Health Organization Trial Registration Data Set items are summarised in Table 1. The SPIRIT Checklist is in the supplementary files (S1 Checklist). This is the first version of the protocol (12 February 2024). There will be no financial compensation for participation in the SphingoFIT study.

**Table 1:** Items from the World Health Organization trial registration data set.

Data category	Information
Primary registry and trial identifying number	Clinicaltrials.gov, NCT06024291
Date of registration in primary registry	August 28, 2023
Secondary identifying numbers	SNCTP000005814, BASEC2023-01345
Source(s) of monetary or material support	Swiss National Science Foundation
	Research Fund of the University of Basel for Excellent Junior Researchers
	Swiss Life Jubilee Foundation
Primary sponsor	Prof Arno Schmidt-Trucksäss, MD
Secondary sponsor(s)	n/a
Contact for public queries	Dr Justin Carrard, MD, Justin.carrard@unibas.ch
Contact for scientific queries	Dr Justin Carrard, MD, Justin.carrard@unibas.ch
Public title	The SphingoFIT Study
Scientific title	Effect of an eight-week high-intensity interval training programme on circulating sphingolipid levels in middle-aged adults at elevated cardiometabolic risk (SphingoFIT)–Protocol for a randomised controlled exercise trial
Countries of recruitment	Switzerland
Health condition(s) or problem(s) studied	Physiological response of sphingolipids to an eight-week high-intensity interval training programme
Intervention(s)	Intervention group: three high-intensity interval training sessions per week for eight weeks
	Control group: physical activity counselling at baseline based on current guidelines
Key inclusion and exclusion criteria	Ages eligible for study: 40–60 years
	Sexes eligible for study: both
	Accepts healthy volunteers: no
	Inclusion and exclusion criteria: see Table 2
Study type	Interventional
	Allocation: randomised
	Intervention model: parallel assignment
	Masking: double (up to the intervention only)
	Primary purpose: prevention
Date of first enrolment	November 17, 2023
Target sample size	98
Recruitment status	Recruiting and data collecting
Primary outcome(s)	Concentration of circulating Cer16:0, Cer18:0, Cer24:0 and Cer24:1
Key secondary outcomes	Concentration of the resting circulating sphingolipid species to be acquired,
	Cardiorespiratory fitness (VO2peak),
	Cholesterol (total-, high-density lipoprotein- and low-density lipoprotein-), triglycerides, glycated haemoglobin, the homeostatic model assessment for insulin resistance,
	Static retinal vessel analysis,
	Flow-mediated dilatation,
	Strain analysis of the heart cavities.

### Recruitment and eligibility criteria

This randomised controlled trial aims to include 98 participants (50% of females) aged between 40 and 60 years at elevated cardiometabolic risk. Participants meeting the inclusion criteria (Table 2) will be eligible for the study. To investigate whether circulating sphingolipid levels can be reduced following an 8-week HIIT intervention, including patients at elevated cardiometabolic risk but without symptomatic cardiometabolic diseases is ideal. Indeed, these patients are likely situated in the first phase of sphingolipid accumulation, which is believed to be reversible.

**Table 2:** Inclusion and exclusion criteria.

Inclusion criteria	Exclusion criteria
• female or male sex • aged between 40 and 60 years • body mass index between 25.0 and 34.9 kg/m^2^ • sedentary lifestyle, defined as not meeting the WHO guidelines on PA, i.e., at least 150 minutes of moderate-intensity aerobic PA per week as well as muscle-strengthening activities on two or more days per week [28] • medical clearance for HIIT by a study physician (including vital sign evaluation, clinical examination, resting and exercise ECG) • informed consent as documented by signature	• known pregnancy or breastfeeding • any current exercise-limiting musculoskeletal conditions of the lower limbs • any known current or chronic conditions limiting exhaustive exercise • known diabetes mellitus of any type • dyslipidaemia, if pharmaceutically treated • arterial hypertension ≥160/100 mmHg, pharmaceutically treated or not • any other known cardiovascular disease • known NASH • known macular degeneration, glaucoma, or high intraocular pressure (≥20 mm Hg) • particular diet (vegetarian, vegan, lactose-free, gluten-free, or FODMAP-low diet (fermentable oligosaccharides, disaccharides, monosaccharides, and polyols) • intake of glucagon-like peptide-1 analogues, orlistat or any weight-loss drug • inability to follow the procedures of the study, e.g., due to linguistic or cognitive problems • concomitant involvement in another interventional trial or participation in another interventional trial in the last four weeks

Inhabitants from the Basel area will be invited to participate in this study via advertisements in local newspapers and newsletters and on diverse social media channels. Detailed information on study procedures, risks, and benefits will be provided to participants by a study physician over the phone. They will also be informed about their right to withdraw from the study without consequences or the need to provide reasons. Following the phone call, all potential participants will be provided via e-mail with a participant information sheet and a consent form. They will be given at least 24 hours (after receiving the participant information sheet and consent form) to decide whether to participate. Formal written consent will be obtained before the participant is submitted to any study procedure. The recruitment will be continuous until the targeted 98 participants are enrolled.

### Group allocation and randomisation

After the baseline examination, participants (n = 98, 50% females) will be randomly allocated to the intervention or the control group. Blocked randomisation will be used to reduce bias and balance participant allocation to both groups [49]. The randomisation process will be stratified by age and sex to ensure a balanced allocation of these variables between both groups. The randomisation code can be found here: https://github.com/JustinCarrard/SphingoFIT. The randomisation will be done by a statistician not involved in recruitment or data collection and will be revealed to participants and the study staff after the baseline examination. Therefore, participants, exercise scientists, and physicians supervising the intervention will be blinded for group allocation at baseline but not post-intervention.

### Study procedure

**Fig 2:**
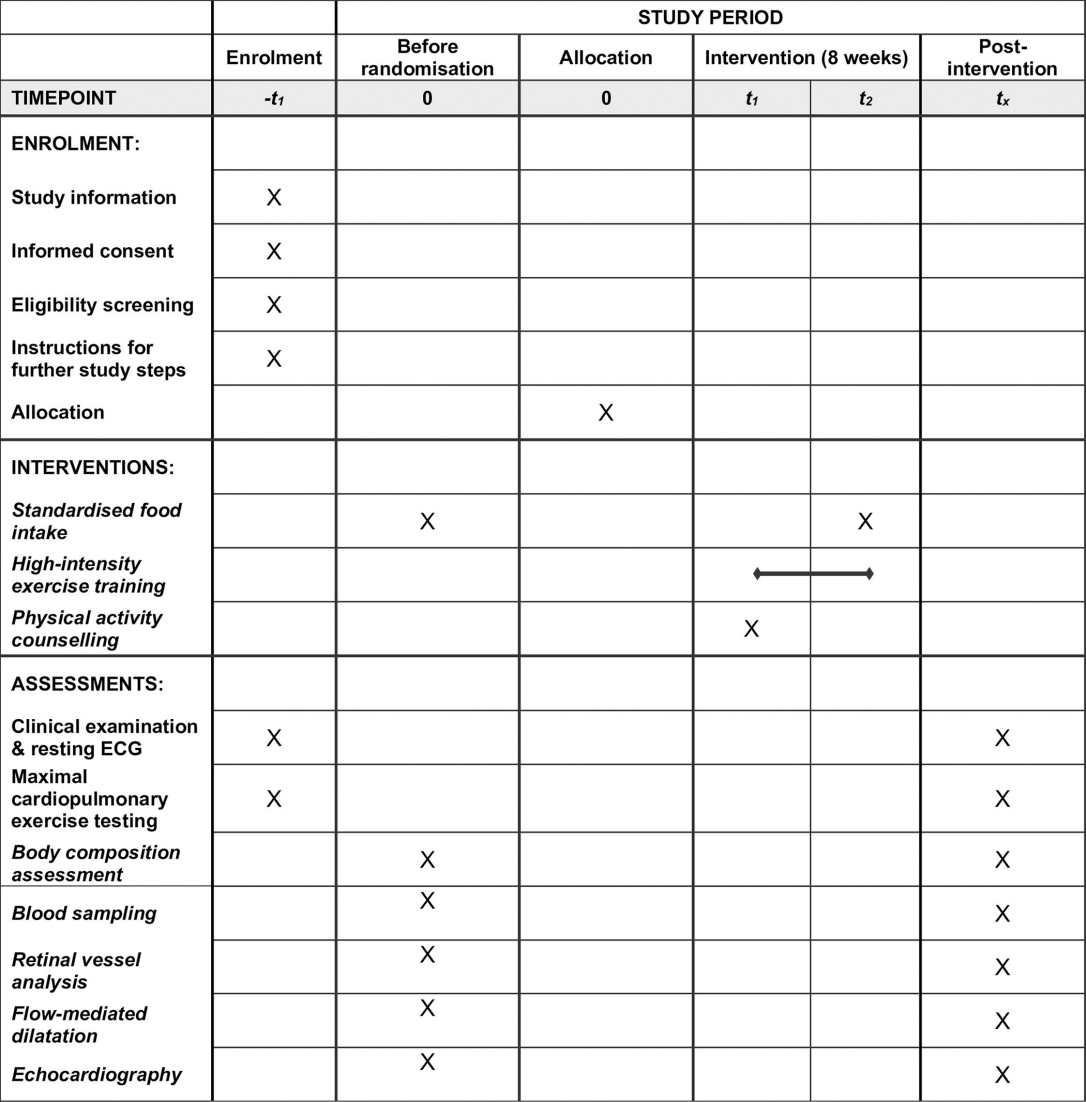
Standard Protocol Items: Recommendations for Interventional Trials (SPIRIT) schedule of enrolment, interventions, and assessments.

**Fig 3:**
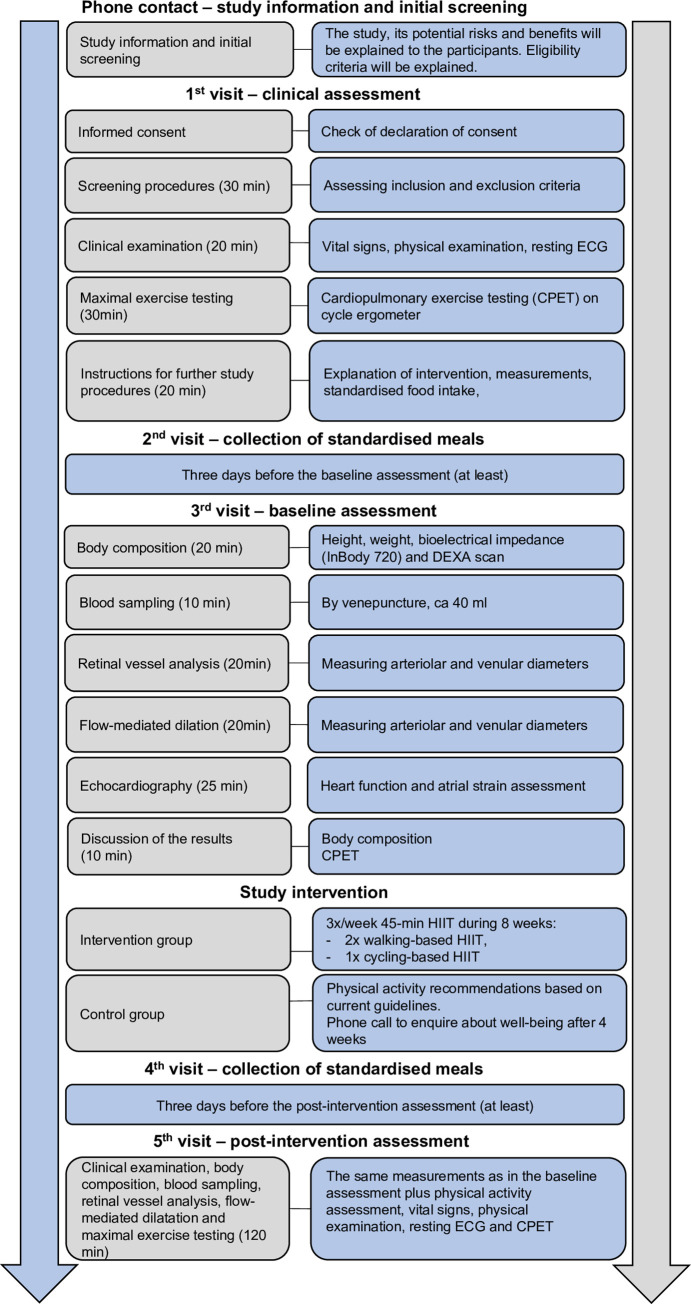
Schedule and sequence of study procedures as planned for study visits. Abbreviations: ECG = electrocardiogram, CPET = cardio-pulmonary exercise testing, HIIT = high intensity interval training.

The SPIRIT schedule of enrolment, interventions, and assessments are summarised in Fig 2 and complemented by Fig 3.

### Eligibility screening

Screening for eligibility will be performed by phone, including the inclusion and exclusion criteria assessment. Participants who pass this screening will be invited to a clinical evaluation at the Department of Sport, Exercise and Health of the University of Basel.

#### 1st visit–clinical and eligibility assessment

A clinical assessment will be conducted, which includes vital sign evaluation (blood pressure, heart rate, respiration rate, oxygen saturation, and body temperature) and physical examination (focusing on cardiovascular and respiratory systems). Height and body weight will be measured to the nearest 0.5 cm and 0.1 kg, respectively, and the body mass index will be calculated. A resting and exercise ECG will be part of the clinical assessment to screen for cardiac contraindications to maximal exertion. Habitual PA will be assessed using the Global Physical Activity Questionnaire [50], validated in a Swiss context [51]. Maximal cardiopulmonary exercise testing (CPET) will be conducted on a cycle ergometer to determine VO_2_peak, peak heart rate and power output (Ergoselect 200; Ergoline, Bitz, Germany; MetaMax 3B; Cortex Biophysik GmbH, Leipzig, Germany). Finally, medical clearance will be given during this first visit. Participants successfully passing this screening will be invited for a baseline assessment. Instructions for the further procedures of the study will be provided.

#### 2nd visit–collection of standardised meals

To our knowledge, no evidence exists that food intake influences sphingolipid levels. Nevertheless, each participant will receive standardised, pre-packaged meals for the two days preceding blood sampling to minimise potential confounding. All participants will be fed to energy balance. Energy requirements will be calculated using the formulas of Mifflin St. Joer [52] and the National Institute of Diabetes and Digestive and Kidney Diseases Body Weight Planner [53, 54]. All diets will contain ~55% energy from carbohydrates, ~25% from fat, and ~20% from protein. To monitor diet adherence, participants will be instructed to return all non-consumed foods from the pre-packaged meals to the lab and take photos of additionally consumed foods for later analysis. Participants will also be asked to refrain from alcohol consumption during the dietary control period (i.e., the two days preceding blood sampling). Participants will be advised to eat as usual for the rest of the study.

Each participant will collect, at least three days before the baseline assessment, their standardised, pre-packaged meals for the two days preceding blood sampling. Depending on the delay between the first visit and the baseline assessment, this second visit can be combined with the first visit.

#### 3rd visit–baseline assessment

Trained medical staff will draw blood samples by venepuncture of the cubital fossa following an overnight fast pre-and post-intervention. The total volume of blood samples will be ca. 18 mL (1 × 2.7 mL potassium-EDTA, 1 × 7.5 mL serum-monovette, and 1 × 7.5 mL Li-heparin). Potassium-EDTA samples will be slightly shaken by hand before being frozen at − 80°C. To ensure complete coagulation, serum samples will be slightly shaken on a shaking platform for 10 min. Within a maximum of 30 min following blood sampling, serum and plasma samples will be centrifuged (at 2,000 g, that is, 4,500 rpm for 10 min at 20°C). Serum and plasma samples will be aliquoted, and aliquots will be frozen at − 80°C. Planned blood analyses for essential characterisation of risk factor profiles include total cholesterol, LDL- and HDL-cholesterol, triglycerides, and HbA1c. Glucose and insulin will also be measured to estimate insulin resistance using the Homeostatic Model Assessment for Insulin Resistance (HOMA-IR) [55].

Body composition will be analysed before and after the intervention by the gold-standard, dual-energy x-ray absorptiometry (DXA) using a GE Lunar iDXA® machine (GE Lunar Inc., Madison, WI; software version 13.10). Study participants will fast within 6 hours before the measurement. Absolute and relative measures of total body fat mass and fat-free mass will be obtained. During the measurement, patients will be in a supine position [56]. The effective radiation dose from a single whole-body DXA (< 10 μSv) is similar to the normal background radiation received over one day at sea level [57]. Consequently, the effective radiation dose per study participant in the present study will be < 20 μSv, similar to the normal background radiation received over two days at sea level. For comparison purposes, body composition will also be analysed before and after the intervention by four-segment bioelectrical impedance analysis using the InBody 720 (Inbody Co. Ltd., Seoul, South Korea). Before the measure, participants must refrain from intense PA for 24 hours, fast for at least two hours, and be asked to void their bladder.

Retinal vessel diameters, a novel surrogate of microvascular health that responds positively to exercise intervention, will be assessed pre- and post-intervention as described in detail by Hanssen et al. [58]. Arteriolar and venular diameters will be measured using semiautomatic software based on a modified fundus camera (450FF; Carl Zeiss, Jena, Germany). Three valid images of each retina will be taken at an angle of 45° and with the optic disc in the centre. Retinal arterioles and venules within a distance of 0.5- to 1.0-disc diameter from the optic disc will be identified semi-automatically (Vesselmap 2; IMEDOS Systems, Jena, Germany). Vessel diameters will be averaged to central retinal arteriolar (CRAE) and venular equivalents (CRVE), as well as their ratio, using the Parr–Hubbard formula described elsewhere [59]. This reliable method has inter- and intra-observer interclass correlation coefficients between 0.78 and 0.99 [59, 60].

Brachial artery flow-mediated dilatation (FMD) reflects endothelial function as an early marker of atherosclerotic arterial damage [61]. It provides an in vivo, non-invasive, direct measure of artery function and health [62]. There is increasing evidence that FMD in humans is associated with traditional risk factors, cardiovascular diseases, and heart failure and predicts cardiovascular events and all-cause mortality [61, 63]. It is sensitive to changes in behavioural health shown for the Mediterranean diet, acute mental stress, or exercise training [64–66]. Measurement of FMD of the brachial artery follows at least 15-20min of rest in the supine position using a high-resolution linear ultrasound array transducer with a semi-automatic detection program of the intima-media complex (EF, Unex Corporation, Nagoya, Japan). An occlusion cuff will be wrapped around the forearm with the proximal edge of the cuff at the elbow. Longitudinal images of the right brachial artery (typically at 3–5 cm above the elbow) are recorded at baseline and after cuff deflation following supra systolic compression (50 mmHg over the systolic blood pressure value) of the right forearm for 5 minutes until 3 minutes after deflation. Inter-reader and inter-session reliability of the FMD measurement were acceptable [67, 68].

Echocardiographic assessment, particularly atrial strain analysis—an emerging ultrasound-based technique—facilitates the comprehensive evaluation of atrial mechanics, offering invaluable insights into atrial fibrosis [69]. Atrial fibrosis, in turn, is a hallmark of atrial structural remodelling, which leads to atrial fibrillation, the most common cardiac arrhythmia with an ever-increasing prevalence [70, 71]. Strain analysis, a highly sensitive measure of cardiac function, has demonstrated responsiveness to even short periods of endurance exercise training, as observed in a 4-week HIIT intervention [72]. Standardised echocardiographic images will be systematically acquired in the supine position, adhering to international standards [73, 74]. The imaging process will use a state-of-the-art ultrasound system (Philips Epiq 7 Ultrasound System, Koninklijke Philips N.V., Amsterdam) equipped with a compatible broadband sector array transducer (S5-1, Philips, Koninklijke Philips N.V., Amsterdam). Post-acquisition, echocardiographic images will undergo meticulous analysis for strain parameters within the ultrasound workspace, utilising advanced software (TOMTEC Imaging Systems GmbH, Unterschleissheim, Germany).

#### Training intervention

The exercise intervention will consist of a supervised HIIT (two walking- and one indoor cycling-based session weekly), starting with a habituation week at an intensity of 75% of the maximal heart rate (HRmax). In the following seven weeks, the participants will perform a HIIT based on the following protocol and for a total duration of 45 min per session (modified from Wisløff et al. [75]): warm-up for 10 min at 60%–70% HRmax followed by a high-intensity interval consisting of 4×4 min at 80%–95% HRmax with 3 min of active recovery at 60%–70% HRmax and a 10 min cool-down at 60%–70% HRmax. Heart rate will be monitored during training by Garmin HRM-Dual heart rate sensors combined with Garmin Forerunner 45S watches. Exercise scientists motivate the participants during the intervals and will control each participant’s heart rate during and after every training session. This protocol was chosen because it fulfils the requirements of a high-volume HIIT [76], has been extensively studied in both healthy and clinical populations, and its effects on CRF improvement are well-documented [39, 77]. In addition, it has been previously used by our research group, with high adherence and absence of drop-out observed in healthy participants and patients with cardiovascular risk factors [78, 79].

#### Control condition

Asking physically inactive participants to maintain inactive habits may not reflect realistic conditions and is no longer considered the best option in a randomised controlled exercise intervention [80]. Indeed, most participants will be aware of the positive effects of PA or will become aware of them during an exercise intervention study [80]. Further, denying an exercise intervention to participants who would have benefited from it (for instance, participants at risk of cardiometabolic diseases) might be ethically questionable [80]. Following current practices [80], control group participants will be informed about the World Health Organization (WHO) PA guidelines at the beginning of the study [81]. After four weeks, the control group participants will get a phone call to enquire about their well-being.

#### 4^th^ visit–collection of standardised meals

Each participant will collect, at least three days before the baseline assessment, their standardised, pre-packaged meals for the two days preceding blood sampling at post-intervention assessment. This visit can be combined with a training session for the intervention group.

#### 5th visit–post-intervention assessment

**Fig 4:**
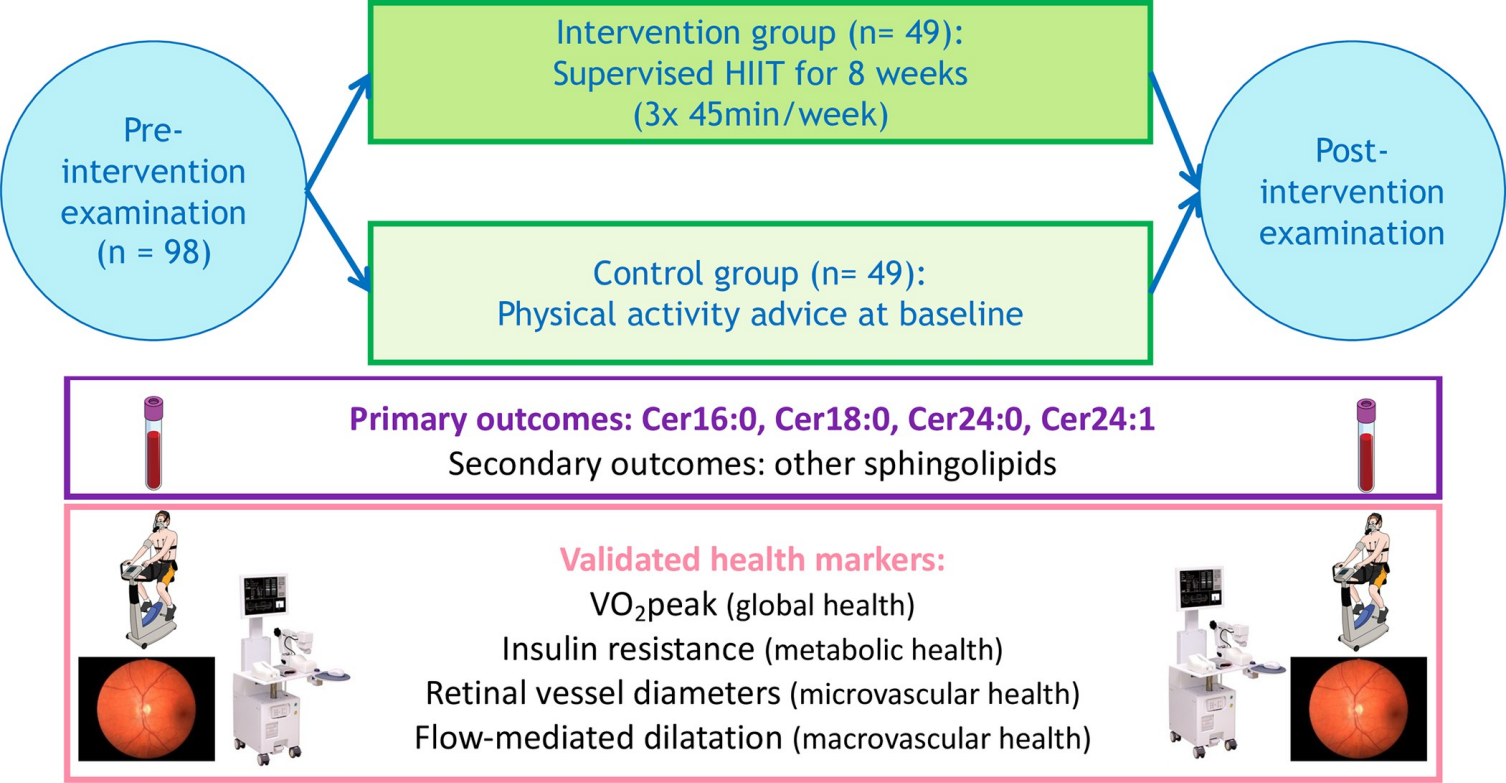
Study design. Abbreviations: HIIT = high intensity interval training, Cer = Ceramide, VO_2_peak = peak oxygen uptake.

After completing the 8-week intervention, the same measurements from the baseline assessment will be repeated (including vital sign evaluation, clinical examination, height and weight measurement, BMI calculation, resting and exercise ECG). A second CPET will be performed to verify that the exercise intervention effectively improved CRF. Habitual PA will be re-assessed using the Global Physical Activity Questionnaire [50, 51]. Fig 4 summarises the study design.

### Sphingolipid quantification

All plasma samples, stored at -80°C at the Department of Sport, Exercise and Health of the University of Basel until the end of the study, will be delivered on dried ice to the Metabolomics Unit of the Faculty of Biology and Medicine at the University of Lausanne.

Plasma samples, calibrators, and quality controls (QCs, 25μL) will be extracted by the addition of 100μL of methanol spiked with internal standards in 96 deep-well plates (Waters, Milford, MA, USA) using a Bravo automated liquid-handling platform (Agilent Technologies, Santa Clara, California, USA). The extracts will be shaken (10 min at 1000 rpm) and centrifuged externally (15 min at 2700g at 15°C) (Hermle Z 326 K, Gosheim, Germany). The liquid handler will transfer the resulting supernatant (75 μL) into a new 96-well plate (Thermo Scientific, San Jose, CA, United States) for the injection. Sphingolipid quantification will be performed by reversed-phase liquid chromatography coupled to tandem mass spectrometry (RPLC-MS/MS) in positive ionisation mode, using a TSQ Altis triple-stage quadrupole mass spectrometer (Thermo Scientific, San Jose, CA, United States) interfaced with Vanquish™ Duo UHPLC System, adapted from Checa et al. [82]. Briefly, the chromatographic separation is carried out on a Zorbax Eclipse plus C18 column (1.8 μm, 100 mm × 2.1 mm I.D) (Agilent Technologies, Inc.). The mobile phase comprises A = 5 mM ammonium formate and 0.2% formic acid in water and B = 5 mM ammonium formate and 0.2% formic acid in MeOH. The flow rate will be 600 μL/min, column temperature at 40°C, and sample injection volume at 4μl. Gradient elution starting at 75% of B will be linearly increased to 100% over 11 min and held at 100% until 17 min. The column will then be equilibrated to initial conditions for 3 minutes. Optimised HESI source parameters will be set as follows: spray voltage 3500 V, Sheath Gas (Arb) = 60, Aux Gas (Arb) = 15, Sweep Gas (Arb) = 1 and Ion Transfer Tube Temperature 380°C. Nitrogen will be used as the nebuliser, and argon will be used as the collision gas (1.5mTorr); the vaporiser temperature will be set at 350°C. Optimised compound-dependent parameters will be used for data acquisition in timed-selected Reaction Monitoring (t-SRM) mode. Raw data files will be processed using TraceFinder Clinical Research 4.1 software (Thermo Fisher Scientific). Calibration curves and the stable isotope-labelled internal standards (IS) will be used to determine the response factor for absolute quantification. The linearity of calibration curves will be evaluated for each species using a 12-point range; peak area integration will be manually curated and corrected when necessary. Reported sphingolipid species will be named according to the LIPID MAPS classification and nomenclature systems [83–86].

### Sample size calculation

**Fig 5:**
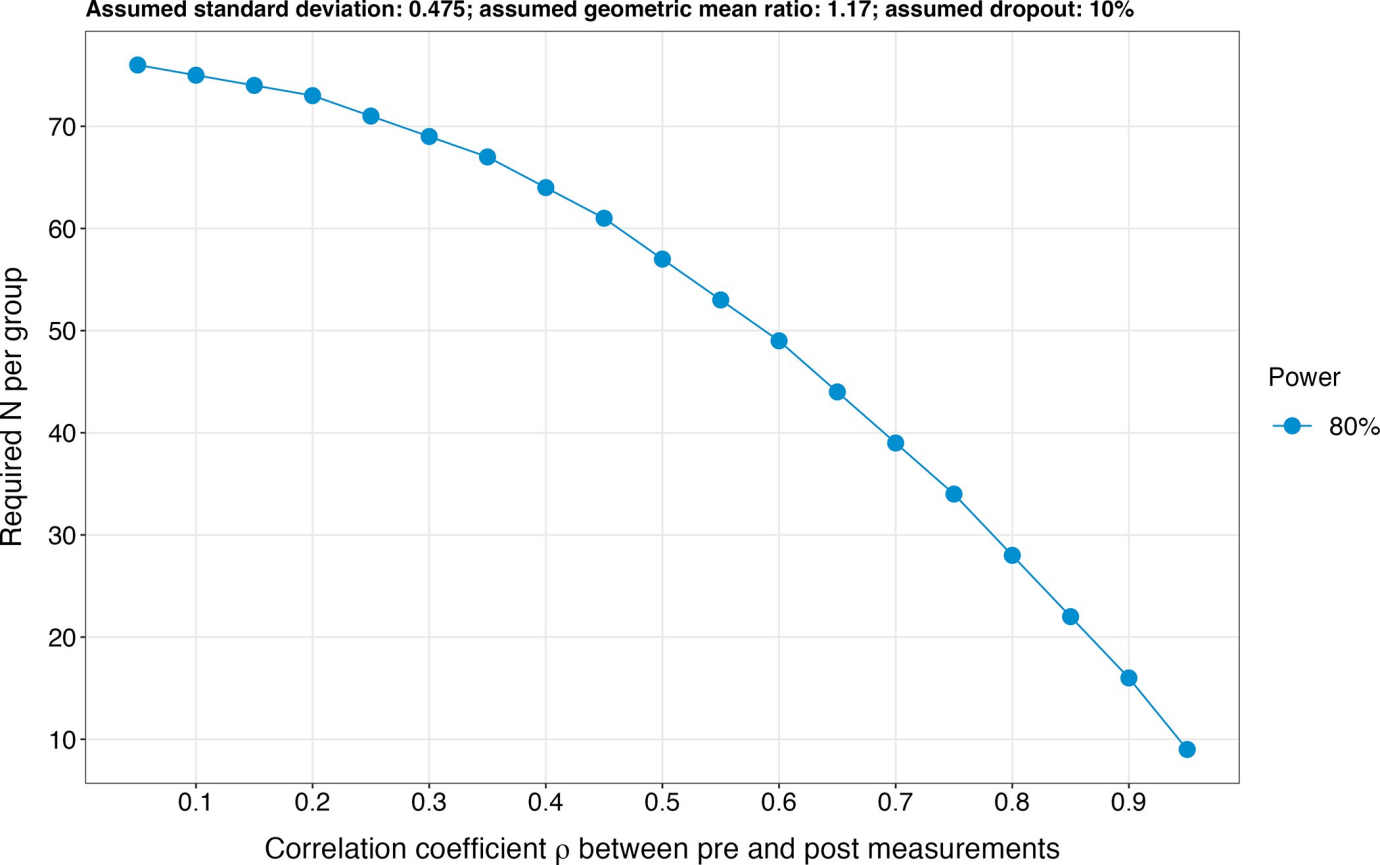
Sample size calculation.

The sample size calculation is based on the four primary endpoints, which are also the species entering the ceramide score (i.e., ceramide 16:0, ceramide 18:0, ceramide 24:0, and ceramide 24:1) [24, 25]. It was hypothesised that 1) log2-transformed pre-intervention sphingolipid levels are similar in both the intervention and the control groups and 2) the standard deviation of log2-transformed sphingolipid levels correspond to 0.475 (which is the average standard deviation obtained for the four sphingolipids mentioned above in participants of the COmPLETE Health study aged 40–60 years [87]). As the results of the SphingoHIIT study are not available yet, the effect size was estimated based on the unique intervention study available in the literature [35]. The authors of this study reported an average effect size (expressed as a geometric mean ratio) of 1.17. This means that the geometric mean of the sphingolipid levels was 1.17 times higher in the control than in the intervention group following the exercise intervention. Assuming a realistic correlation coefficient ρ of 0.6 between pre-and post-intervention values for sphingolipid levels, an analysis of covariance (ANCOVA) was used to calculate the sample size. A result of 49 participants per group for a power of 80% was obtained (Fig 5). Notably, a drop-out rate of 10% was considered in the sample size calculation [80]. The sample size calculation code can be found here: https://github.com/JustinCarrard/SphingoFIT.

### Statistical analyses

**Fig 6:**
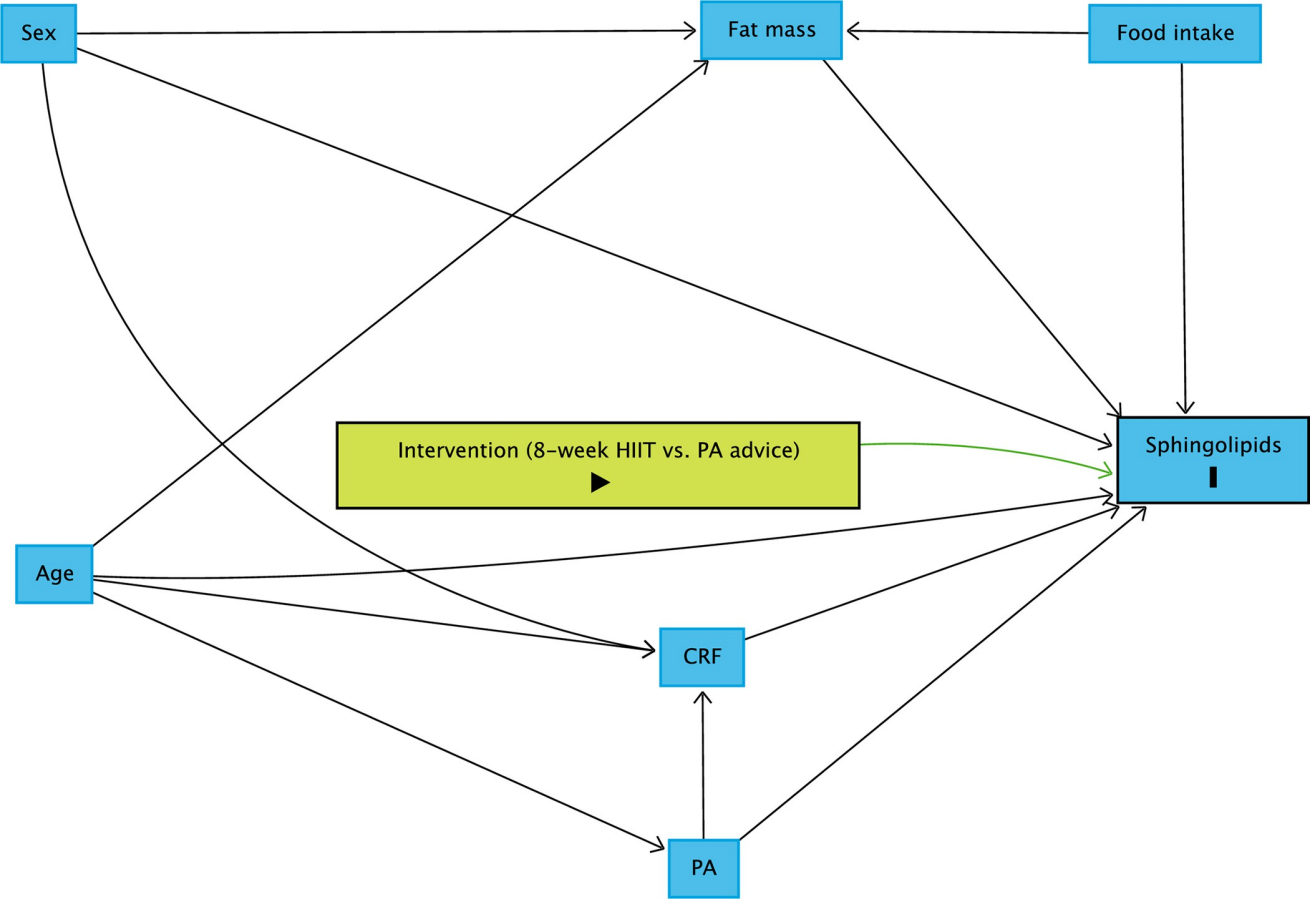
Directed acyclic graph representing the effect of HIIT on sphingolipid levels and other influencing variables. Abbreviations: HIIT = high-intensity interval training, CRF = cardiorespiratory fitness, PA = physical activity, SL = sphingolipid levels.

An ANCOVA will be run for each sphingolipid species to estimate the effect of the intervention [88]. The post-intervention sphingolipid value will be the dependent variable. In contrast, the pre-intervention sphingolipid value, a group variable (i.e., intervention or control group) and all control variables will be the independent variables. To identify control variables to be included in the models, we drew a causal-directed acyclic graph (DAG) using DAGitty [89]. Age, sex, body fat mass and CRF were identified as variables to be included in the models to reduce the outcome variation and improve the precision of the average causal effect of the intervention (Fig 6) [90]. The design will control food intake (each participant will receive individualised, pre-packaged meals for preceding blood sampling) and PA. Graphical methods will be used to assess the normal distribution of data. If the data are not normally distributed, sphingolipid concentrations will be log-transformed. A complete case analysis will be done if the proportion of missing data is below 5%. Otherwise, we will consider multiple imputations. The Benjamini-Hochberg method will adjust P-values for multiple testing [91]. The significance level is set at α = 0.05; all tests will be two-sided. All analyses will be done according to the intention-to-treat principle. Statistical analyses will be conducted using R (version 4.0.2 or later). The study will report its results in compliance with the CONSORT statement [92].

### Data management

Data will be pseudonymised, stored on the protected server of the University of Basel, and accessible only to authorised personnel to fulfil the research objectives described in the present protocol. Biological material collected during the SphingoFIT study will be stored at the Department of Sport, Exercise and Health for ten years after study termination. The data management plan is available in the supplementary files (S2 File).

### Safety considerations

Any adverse event will be classified, and its severity assessed by the investigator according to the guidelines of the International Council for Harmonisation of Technical Requirements for Pharmaceuticals for Human Use [93]. The Ethics Committee will be informed in due time as required by Swiss law.

Exercise is considered a safe approach to optimise cardiometabolic health. The risk for cardiovascular events through exercise is reduced by performing a resting and an exercise ECG before starting the intervention to screen for pathological changes in the heart. The training load is divided between walking- and cycling-based sessions to alternate load and minimise the risk of musculoskeletal injury. Thus, the potential benefits of gaining fitness and thus improving different health parameters outweigh these risks. All subjects also benefit from receiving personal health information and expensive and complex examinations that can give them detailed information about their fitness status.

### Study status

The recruitment started on November 1, 2023, and is still ongoing. Recruitment is estimated to be finished by the end of October 2024.

### Amendments

As required by Swiss law, substantial changes to the study setup and organisation, the protocol and relevant study documents will be submitted to the Ethics Committee of Northwest and Central Switzerland for approval before implementation. Under emergency circumstances, deviations from the protocol to protect the rights, safety, and well-being of human subjects may proceed without prior approval of the Ethics Committee of Northwest and Central Switzerland. Such deviations shall be documented and reported to the Ethics Committee as soon as possible. A list of all non-substantial amendments will be submitted once a year to the Ethics Committee of Northwest and Central Switzerland.

On January 19, 2024, an amendment was submitted to the Ethics Committee of Northwest and Central Switzerland to include an echocardiographic assessment of the participants, which will be included in the study after February 26, 2024. This will enable strain analysis of heart cavities in an explorative manner. This amendment was approved by the Ethics Committee of Northwest and Central Switzerland on January 23, 2024 (EKNZ 2023–01345).

## Discussion

Sphingolipids, in general, and ceramides, in particular, are now recognised as robust and clinically useful predictors of cardiometabolic risk [15, 16, 94]. A score based on the circulating level of four ceramide species has been developed and validated. It is now used in clinical in Finland and at the Cleveland and Mayo Clinic [24, 25]. However, little is known about the possibilities of lowering sphingolipid levels. If circulating sphingolipids are to be measured in daily clinical routines, it is key to provide patients with evidence-based interventions that can reduce sphingolipid levels. While exercise interventions seem ideal candidates for mitigating sphingolipid levels [26], only one unpowered preliminary study examined the effectiveness of a 12-week moderate-intensity continuous training to lower the circulating level of sphingolipids [35]. Yet it has been demonstrated that HIIT is a safe [36, 37] and a more effective way to improve cardiometabolic health in healthy individuals and patients with cardiometabolic diseases [38–40]. Therefore, there is a real need to conduct this powered randomised control trial to inform clinicians whether and to what extent a fitness-enhancing HIIT-based training programme can lower circulating sphingolipid levels. Findings from the study could also contribute to reducing the gap in healthcare between low-income and high-income countries, as HIIT training is feasible in low-income countries due to its practicality and low cost. Finally, this study will also deepen the molecular understanding of exercise medicine and contribute to establishing this novel medical discipline as a cornerstone in the prevention and treatment of cardiometabolic disorders.

### Data dissemination

Findings and data will be disseminated in scientific journals and meetings. Authorship will respect the recommendation of the International Committee of Medical Journal Editors (https://icmje.org/recommendations/browse/roles-and-responsibilities/defining-the-role-of-authors-and-contributors.html).

## Supporting information

S1 Checklist SPIRIT checklist of the SphingoFIT study. (DOC)

S1 File (PDF)

S2 File Data management plan of the SphingoFIT study. (PDF)
