# Supplementary material for: Effect of an eight-week high-intensity interval training programme on circulating sphingolipid levels in middle-aged adults at elevated cardiometabolic risk (SphingoFIT)—Protocol for a randomised controlled exercise trial
Source: PLoS One. 2024 May 8;19(5):e0302477. doi: 10.1371/journal.pone.0302477 (PMC11078397; doi:10.1371/journal.pone.0302477)
Supplement: S1 File — (PDF) [file pone.0302477.s002.pdf]

Reducing Circulating Sphingolipid Levels to Optimise Cardiometabolic Health - The SphingoFIT Randomised Controlled Exercise Trial

Study Type:	Other Clinical Trial according to ClinO, Chapter 4
Risk Categorization:	Risk category A according to https://www.kofam.ch/de/categoriser
Study Registration:	Will be registered in ClinicalTrials.gov and FOPH portal SNCTP (Swiss National Clinical Trial Portal)
Sponsor:	Prof. Dr Arno Schmidt-Trucksäss, MD, Division of Sport and Exercise Medicine, Department of Sport, Exercise and Health of the University of Basel
Principal Investigator	Dr Justin Carrard, MD, Division of Sport and Exercise Medicine, Department of Sport, Exercise and Health of the University of Basel
Investigated Intervention:	8-week high-intensity interval training programme
Protocol ID	2023-01345
Version and Date:	Version 3 (dated 19/01/2024)

CONFIDENTIALITY STATEMENT

The information contained in this document is confidential and the property of the principal investigator, Dr Justin Carrard. MD. The information may not - in full or in part - be transmitted, reproduced, published, or disclosed to others than the applicable Competent Ethics Committee and Regulatory Authorities without prior written authorisation from the principal investigator except to the extent necessary to obtain informed consent from those who will participate in the study.

PROTOCOL SIGNATURE FORM

Study Title	Reducing Circulating Sphingolipid Levels to Optimise Cardiometabolic Health - The SphingoFIT Randomised Controlled Exercise Trial
Study ID	2023-01345

The Sponsor has approved the protocol version 3 (dated 19/01/2024) and confirms hereby to conduct the study according to the protocol, the current version of the World Medical Association Declaration of Helsinki, and ICH-GCP guidelines as well as the local legal applicable requirements.

Sponsor:

Name: Prof. Dr Arno Schmidt-Trucksäss, MD, Division of Sport and Exercise Medicine, Department of Sport, Exercise and Health of the University of Basel

Date: 19/01/2024

Signature:

Principal Investigator:

Name: Dr Justin Carrard, MD, Division of Sport and Exercise Medicine, Department of Sport, Exercise and Health of the University of Basel

Date: 19/01/2024

Signature:

STUDY SYNOPSIS

**Sponsor / Sponsor-Investigator**	Prof. Dr. Arno Schmidt-Trucksäss, MD<br>Department of Sport, Exercise and Health, University of Basel<br>Grosse Allee 6, 4052 Basel<br>E-Mail: arno.schmidt-trucksaess@unibas.ch
**Study Title:**	Reducing Circulating Sphingolipid Levels to Optimise Cardiometabolic Health - The SphingoFIT Randomised Controlled Exercise Trial
**Short Title / Study ID:**	The SphingoFIT Study
**Protocol Version and Date:**	Version 3, 19.01.2024
**Trial registration:**	This trial will be registered in the clinicaltrials.gov database and at the Swiss National Clinical Trials Portal.
**Study category and Rationale**	Clinical Trial according to ClinO, Chapter 4<br>Risk category A according to https://www.kofam.ch/de/categoriser<br><br>The SphingoFIT study is a clinical trial that encompasses an intervention that is neither a trial involving drugs or transplant products nor a trial of transplantation.
**Clinical Phase:**	Not applicable
**Background and Rationale:**	Cardiometabolic diseases (CMD) account for about half of all deaths from non-communicable diseases and are responsible for about one-third of all deaths worldwide. To combat the growing burden of CMD on health systems, a shift towards more effective prevention and early detection of these diseases is urgently needed.<br><br>Blood lipids have been used since the middle of the last century to determine the risk of developing CMD. Although classically used biomarkers such as cholesterol and triglycerides provide acceptable risk assessment, there is increasing evidence that sphingolipids, particularly ceramides, may allow improved risk assessment. Mechanistically, there is growing data that sphingolipid accumulations lead to atherosclerosis and insulin resistance.<br><br>To measure circulating sphingolipids in clinical practice, it is essential to provide patients with evidence-based interventions that reduce sphingolipid levels and quantify the reduction expected from such an intervention. Preliminary data suggest that regular physical activity (PA), an effective, low-cost, and patient-empowering means of health optimisation, may reduce sphingolipid levels.
**Objective(s):**	To assess the effects of an 8-week supervised high-intensity interval training (HIIT) program (vs. PA recommendations according to current guidelines) on a comprehensive panel of circulating sphingolipids in middle-aged females and males at elevated cardiometabolic risk.
**Endpoints(s):**	Primary endpoints: to quantify changes in plasma levels of the four sphingolipids included in the ceramide-based score (i.e., ceramide 16:0, ceramide 18:0, ceramide 24:0 and ceramide 24:1) following the intervention (an 8-week HIIT programme vs PA recommendations).<br>Secondary endpoints: to assess the changes in the resting sphingolipids to be targeted (n=57), in the Homeostatic Model Assessment for Insulin Resistance (HOMA-IR), peak oxygen uptake (VO_2peak), flow-mediated dilatation (FMD), retinal vessel analysis, and echocardiographic strain of the heart cavities.
**Study design:**	A prospective 2-arm, monocentric randomised controlled trial

<table>
<tr><td>Inclusion / Exclusion criteria:</td><td><u>Inclusion criteria:</u>
female or male sex,
aged between 40 and 60 years,
body mass index between 25.0 and 34.9 kg/m2,
sedentary lifestyle, defined as not meeting the World Health Organization (WHO) guidelines on PA, i.e., at least 150 minutes of moderate-intensity aerobic PA per week as well as muscle-strengthening activities on two or more days per week,
medical clearance for HIIT by a study physician (including vital sign evaluation, clinical examination, resting and exercise ECG),
informed consent as documented by signature.
<u>Exclusion criteria:</u>
known pregnancy or breastfeeding,
any current exercise-limiting musculoskeletal conditions of the lower limbs,
any known current or chronic conditions limiting exhaustive exercise,
known diabetes mellitus of any type,
dyslipidaemia, if pharmaceutically treated,
arterial hypertension ≥160/100 mmHg, pharmaceutically treated or not,
any other known cardiovascular disease (CVD),
known non-alcoholic steatohepatitis (NASH),
known macular degeneration, glaucoma, or high intraocular pressure (≥20 mm Hg),
vegetarian, vegan, lactose-free, gluten-free, or FODMAP-low diet (fermentable oligosaccharides, disaccharides, monosaccharides, and polyols),
intake of glucagon-like peptide-1 analogues, orlistat or any weight-loss drug,
inability to follow the procedures of the study, e.g., due to linguistic or cognitive problems
concomitant involvement in another interventional trial or participation in another interventional trial in the last four weeks</td></tr>
<tr><td>Measurements and procedures:</td><td>Participants will be randomly allocated to either the intervention or the control group. The exercise intervention will consist of an 8-week supervised HIIT (two walking- and one indoor cycling-based session weekly). The control group will get PA recommendations based on current guidelines.
Maximal cardiopulmonary exercise testing (CPET) will be conducted on a cycle ergometer to determine the VO_2peak, peak heart rate and peak power output. After the 8-week training programme, a second CPET will be performed to verify if the exercise intervention effectively improved CRF. Body composition will be analysed before and after the 8-week intervention by dual-energy x-ray absorptiometry and by bioelectrical impedance analysis.
Trained medical staff will draw blood samples by venepuncture of the cubital fossa following an overnight fast pre-and post-intervention. Planned blood analyses for basic characterisation of risk factor profiles include total cholesterol, low-density lipoprotein cholesterol (LDL) and high-density lipoprotein cholesterol (LDL), triglycerides, and HbA1c. Glucose and insulin will also be measured to estimate insulin resistance using the HOMA-IR. A high-coverage method using reversed-phase liquid chromatography coupled to tandem mass spectrometry (RPLC-MS/MS) will be applied to quantify an extensive panel of circulating sphingolipids (n=61).
Retinal vessel diameters, a novel surrogate of microvascular health that responds positively to exercise interventions, will be assessed pre-and post-intervention, and the brachial artery FMD, which reflects endothelial function as an early marker of atherosclerotic arterial damage.
Echocardiographic images will be acquired by a trained physician. Subsequently, these images will undergo analysis utilising vendor-independent software to assess mechanical alterations in the heart cavities through strain analysis, serving as a surrogate parameter for cardiac fibrosis.
Each participant will receive individualised, pre-packaged meals for the two days preceding blood sampling to minimise potential confounding. All participants will be fed to energy balance. To monitor diet adherence, participants will be instructed to return</td></tr>
</table>

	all non-consumed foods from the pre-packaged meals to the lab and take photos of additionally consumed foods for later analysis.
**Study Product / Intervention:**	Two-arm intervention: A 8-week HIIT program vs. PA recommendations based on current guidelines<br>The exercise intervention will consist of a supervised HIIT (two walking- and one indoor cycling-based sessions weekly), starting with a habituation week at an intensity of 75% of the maximal heart rate (HRmax). In the following seven weeks, the participants will perform a HIIT-based training on the following protocol and for a total duration of 45 min per session: warm-up for 10 min at 60%–70% HRmax followed by a high-intensity interval consisting of 4×4 min at 80%–95% HRmax with 3 min of active recovery at 60%–70% HRmax and a 10 min cool-down at 60%–70% HRmax. Heart rate will be monitored during training by Garmin HRM-Dual heart rate sensors combined with Garmin Forerunner 45S watches. Exercise scientists motivate the participants during the intervals and will control each participant's heart rate during and after every training session.
**Control Intervention**	The control group will get PA recommendations based on current guidelines. Participants of the control group will get a phone call after four weeks to enquire about their well-being.
**Number of Participants with Rationale:**	Participants (n = 98, 50% of females) at elevated cardiometabolic risk aged between 40-60 years will be randomised (stratified by sex) into the intervention or the control group. See item statistical consideration below for sample size justification.
**Study Duration:**	Recruitment will continue until the targeted 98 participants are enrolled. We are planning for a study duration of one year.
**Study Schedule:**	Start of recruitment after approval by the ethics committee and registration of the study at ClinicalTrials.gov (expected in early September 2023). Start of the first studies as soon as written consent has been obtained (expected in late September 2023).
**Investigators:**	<u>Sponsor</u><br>Prof. Dr. Arno Schmidt-Trucksäss, MD<br>Department of Sport, Exercise and Health, University of Basel<br>Grosse Allee 6, 4052 Basel<br>E-Mail: arno.schmidt-trucksaess@unibas.ch<br><br><u>Principal Investigator</u><br>Dr. Justin Carrard, MD<br>Department of Sport, Exercise and Health, University of Basel<br>Grosse Allee 6, 4052 Basel<br>E-Mail: justin.carrard@unibas.ch<br><br><u>Sub Investigators</u><br>Prof. Dr. Henner Hanssen, MD<br>Department of Sport, Exercise and Health, University of Basel<br>Grosse Allee 6, 4052 Basel<br>E-Mail: henner.hanssen@unibas.ch<br><br>Dr. Luisa Prechtl, MD<br>Department of Sport, Exercise and Health, University of Basel<br>Grosse Allee 6, 4052 Basel<br>E-mail:luisa.prechtl@unibas.ch
**Study Centre:**	Department of Sport, Exercise and Health, University of Basel<br>Grosse Allee 6<br>4052 Basel
**Statistical Considerations:**	An ANCOVA will be run for each sphingolipid species to estimate the effect of the intervention. The post-intervention sphingolipid value will be the dependent variable. In contrast, the pre-intervention sphingolipid value, a group variable (i.e. intervention or control group) and all control variables will be the independent variables. To identify control variables to be included in the models, we drew a causal-directed acyclic graph (DAG) using DAGitty. Age, sex, body fat mass and CRF were identified as variables to be included in the models to reduce the outcome variation and improve the precision of the average causal effect of the intervention.

	The design will control food intake as each participant will be provided with individualised, pre-packaged meals for the two preceding blood sampling. Graphical methods will be used to assess the normal distribution of data. If the data are not normally distributed, sphingolipid concentrations will be log-transformed. The Benjamini-Hochberg method will adjust P-values for multiple testing. The significance level is set at $\alpha = 0.05$, and all tests will be two-sided. All analyses will be done according to the intention-to-treat principle. If the proportion of missing data is below 5%, a complete case analysis will be done. Otherwise, we will consider multiple imputations. Statistical analyses will be conducted using R (version 4.0.2 or later). The study will report its results in compliance with the CONSORT statement.
**GCP Statement:**	This study will be conducted in compliance with the protocol, the current version of the Declaration of Helsinki, the ICH-GCP or ISO EN 14155 (as far as applicable) as well as all national legal and regulatory requirements.

Explanation for the inclusion of vulnerable subjects:

No vulnerable subjects were included.

Recruitment procedure:

Inhabitants from the Basel area will be invited to participate in this study via advertisements in local newspapers and on diverse social media channels. Patients of the Exercise Medicine Clinic of the DSBG will also be offered to participate. Screening for inclusion and exclusion criteria will be performed by phone, while informed consent, clinical examination, and medical clearance will take place during the first visit. Eligible participants will be invited for a baseline examination, which will take place before the randomisation. Instructions for the further procedures of the study will be given.

Study procedure with timeline:

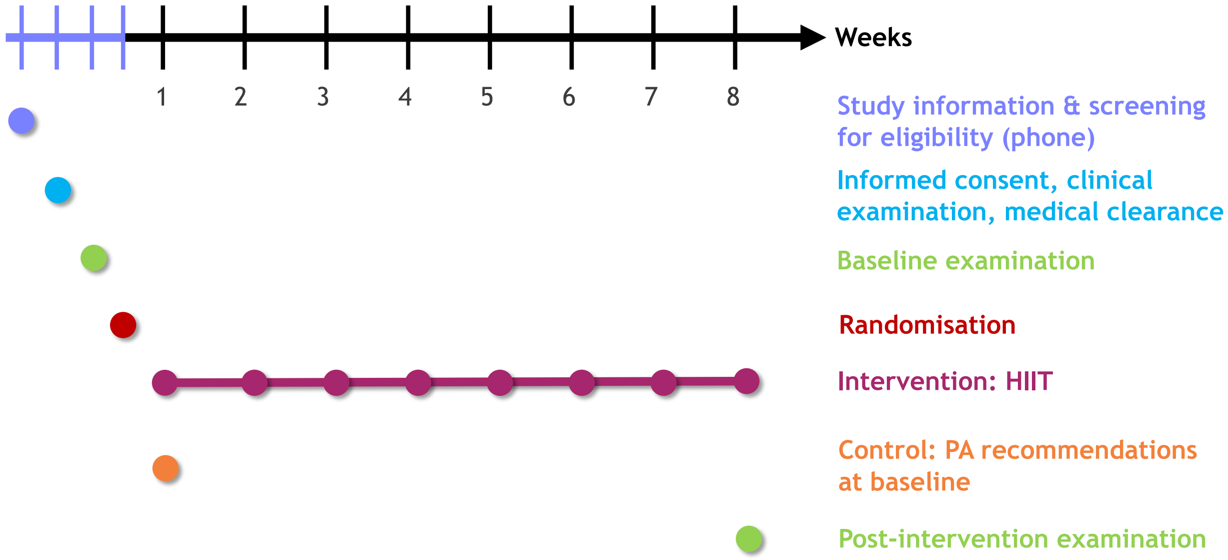

Figure 1: Study timeline

Abbreviations: HIIT = high intensity interval training, PA = physical activity

Risks/inconveniences:

To limit this risk of musculoskeletal injury, the intervention group training will contain two walking-based and one cycling-based session per week to vary the load on the joints.
The risk for cardiovascular events through training is minimised by performing a resting ECG and an exercise ECG before the intervention. In addition, all investigators are trained in basic life support. While doing the maximal exercise testing, a physician and a defibrillator will be on site.

Coverage of damages:

The University of Basel offers general insurance for category A trials.

Storage of data and samples:

Yes, see protocol, section 7.4, page 27.

Ethical considerations:

1. **Please describe the potential gain of new knowledge obtained with this study and its meaning for patients/society.**

Exercise as a prevention method for cardiometabolic diseases is a safe and broadly accessible approach. This study will be another step in generating scientific evidence regarding HIIT training and its influence on the sphingolipidome, leading to new preventive and therapeutic strategies in treating cardiometabolic disease. Positive findings from the study can help reduce the gap in healthcare between low-income and high-income countries, as HIIT training is feasible in low-income countries due to its practicality and low cost.

2. **Please assess the benefit/risk relationship for the patient.**

The SphingoFIT study is designed so the participants can participate in an 8-week training program for free. HIIT brings the risk of short-term exhaustion or muscle soreness. Another posed risk is suffering from a musculoskeletal injury through training. These risks are minimised by varying the training load and integrating two walking-based and one cycling-based session weekly. The risk for cardiovascular events through exercise is reduced by performing a resting and an exercise ECG before starting the intervention. The benefits of improving fitness and thus improving different health parameters outweigh the potential risks of this program. All subjects also benefit by receiving information about their health and fitness status. In addition, their participation helps generate knowledge, forming the basis for new prevention and treatment methods.
All involved parties will keep the participant's data strictly confidential. Data will be entered in protected files on the server of the Department of Sport, Exercise and Health of the University of Basel. Access to these files will be restricted to study group members who agreed on confidentiality.

3. **Please explain why the methodology is also ethically appropriate to gain new generalisable knowledge.**

This is a clinical trial in which the intervention group participants can expect health benefits from participating in the HIIT program. The control group is needed for this trial to demonstrate the effect of the HIIT protocol on circulating sphingolipids. They can also expect a health benefit by giving the control group recommendations for PA based on current guidelines. In addition, both groups will benefit from the thorough exercise and health testing, which provides detailed information about their medical and fitness status.

The most relevant references:

Choi RH, Tatum SM, Symons JD, Summers SA, Holland WL. Ceramides and other sphingolipids as drivers of cardiovascular disease. Nat Rev Cardiol. 2021;18(10):701-11.

Laaksonen R, Ekroos K, Sysi-Aho M, Hilvo M, Vihervaara T, Kauhanen D, et al. Plasma ceramides predict cardiovascular death in patients with stable coronary artery disease and acute coronary syndromes beyond LDL-cholesterol. Eur Heart J. 2016;37(25):1967-76.

Tippetts TS, Holland WL, Summers SA. Cholesterol – the devil you know; ceramide – the devil you don't. Trends Pharmacol Sci. 2021;42(12):1082-95.

Carrard J, Angst T, Weber N, *et al*. Investigating the circulating sphingolipidome response to a single high-intensity interval training session within healthy females and males in their twenties (SphingoHIIT): Protocol for a randomised controlled trial. F1000Res. 2023 Jun 29;11:1565.

1. TABLE OF CONTENTS

1. TABLE OF CONTENTS 8
2. GLOSSARY OF ABBREVIATIONS 9
1. BACKGROUND AND RATIONALE 10
2. STUDY OBJECTIVES AND DESIGN 12
   2.1 Hypothesis and primary objective 12
   2.2 Endpoints 12
   2.3 Study design 12
   2.4 Study intervention 12
3. STUDY POPULATION AND STUDY PROCEDURES 15
   3.1 Inclusion and exclusion criteria, justification of study population 15
   3.2 Recruitment, screening, and informed consent procedure 16
   3.3 Study procedures 17
   3.4 Withdrawal and discontinuation 20
4. STATISTICS AND METHODOLOGY 20
   4.1 Statistical analysis plan and sample size calculation 20
   4.2 Handling of missing data and drop-outs 22
5. REGULATORY ASPECTS AND SAFETY 22
   5.1 Local regulations / Declaration of Helsinki 22
   5.2 (Serious) Adverse Events and notification of safety and protective measures 22
   5.3 (Periodic) safety reporting 23
   5.4 Radiation 23
   5.5 Pregnancy 24
   5.6 Amendments 24
   5.7 Notification and reporting upon completion, discontinuation or interruption of the study 24
   5.8 Insurance 24
6. FURTHER ASPECTS 24
   6.1 Overall ethical considerations 24
   6.2 Risk-benefit assessment 25
7. QUALITY CONTROL AND DATA PROTECTION 25
   7.1 Quality measures 25
   7.2 Data recording and source data 25
   7.3 Confidentiality and coding 25
   7.4 Retention and destruction of study data and biological material 26
8. FUNDING / PUBLICATION / DECLARATION OF INTEREST 26
9. REFERENCES 27

2. GLOSSARY OF ABBREVIATIONS

AE	*Adverse Event*
ASR	*Annual Safety Report*
ANCOVA	*Analysis of Covariance*
BASEC	*Business Administration System for Ethical Committees*
BMI	*Body Mass Index*
ClinO	*Ordinance on Clinical Trials in Human Research (in German: KlinV, in French: OClin, in Italian: OSRUm)*
CMD	*Cardiometabolic diseases*
CPET	*Cardiopulmonary Exercise Testing*
CRF	*Case Report Form*
CRF	*Cardiorespiratory Fitness*
CTCAE	*Common Terminology Criteria for Adverse Events*
CVD	*Cardiovascular diseases*
DAG	*Directed Acyclic Graph*
EC	*Ethics Committee*
ECG	*Electrocardiogram*
FMD	*flow-mediated dilatation*
FODMAP	*Fermentable Oligosaccharides, Disaccharides, Monosaccharides and Polyols*
FOPH	*Federal Office of Public Health*
GCP	*Good Clinical Practice*
GLUT 4	*Glucose transporter type 4*
HDL-C	*High-density lipoprotein cholesterol*
HIIT	*High-intensity interval training*
RPLC-MS/MS	*Reversed-phase liquid chromatography coupled to tandem mass spectrometry*
HRA	*Human Research Act (in German: HFG, in French: LRH, in Italian: LRUm)*
HRmax	*maximal heart rate*
ICH	*International Conference on Harmonisation*
LDL-C	*Low-density lipoprotein cholesterol*
MET	*Metabolic Equivalent of Task*
MICT	*Moderate-intensity continuous training*
NCD	*Noncommunicable diseases*
NIDDK	*National Institute of Diabetes and Digestive and Kidney Diseases*
PA	*physical activity*
PP2A	*Protein phosphatase 2A*
SAE	*Serious Adverse Event*
SNCTP	*Swiss National Clinical Trial Portal*
T2DM	*Type 2 diabetes mellitus*
VO_2peak	*Peak Oxygen uptake*
WHO	*World Health Organization*

1. BACKGROUND AND RATIONALE

Cardiovascular diseases (CVD) are the leading cause of death worldwide and represent a major socioeconomic concern for healthcare systems (1, 2, 3). Early detection and treatment of patients at risk for CVD are crucial to combat this burden effectively (4). However, clinical evaluations do not easily capture the risk of developing CVD, particularly for patients at intermediate risk (5). For instance, low-density lipoprotein (LDL) cholesterol, a pillar of cardiovascular risk assessment, was shown to be elevated in only half of the patients hospitalised with coronary artery disease (CAD) (6). Improving the risk stratification of intermediate-risk patients would enable more individualised primary prevention strategies and eventually reduce the burden related to CVD (5). In the -omics era, there is a real need for novel pathophysiology-based phenotyping tools to improve cardiovascular risk stratification (7).

How sphingolipids drive cardiometabolic diseases

Sphingolipids constitute a family of bioactive lipids which modulate numerous biological processes and are involved in the pathogenesis of CAD, type 2 diabetes mellitus (T2DM), and non-alcoholic fatty liver disease (NAFLD) (8, 9, 10). Sphingolipid-mediated alterations that drive cardiometabolic conditions are illustrated in Figure 1. Briefly, once the triglyceride stores are saturated, in case of overnutrition, reduced energy expenditure or chronic inflammation, lipids in excess are redirected to form sphingolipids (11). In muscle and liver cells mainly, sphingolipids accumulate in the form of ceramides, a particular subclass of sphingolipids (10). Initially, this pathological ceramide accumulation 1) reduces the translocation of glucose transporters to the cell membrane, 2) improves fatty acid storage and uptake, and 3) impairs mitochondrial efficiency, resulting in the production of reactive oxygen species (12). This initial phase of metabolism alteration leads to peripheral insulin resistance and NAFLD (11, 12). Failure to manage this sphingolipid overload results in lipotoxicity, a phenomenon characterised by apoptosis and fibrosis, which lead to CAD, non-alcoholic steatohepatitis (NASH), or T2DM (11, 12). Lastly, ceramides in excess are also carried on LDL, where they drive LDL transcytosis through the endothelium and uptake into macrophages (13, 14). This results in foam cell formation, vascular inflammation, and atherosclerosis (15). Situated at the crossroads of overnutrition, dyslipidaemia and inflammation, sphingolipid metabolism offers a unique opportunity to improve cardiometabolic risk stratification (9, 16).

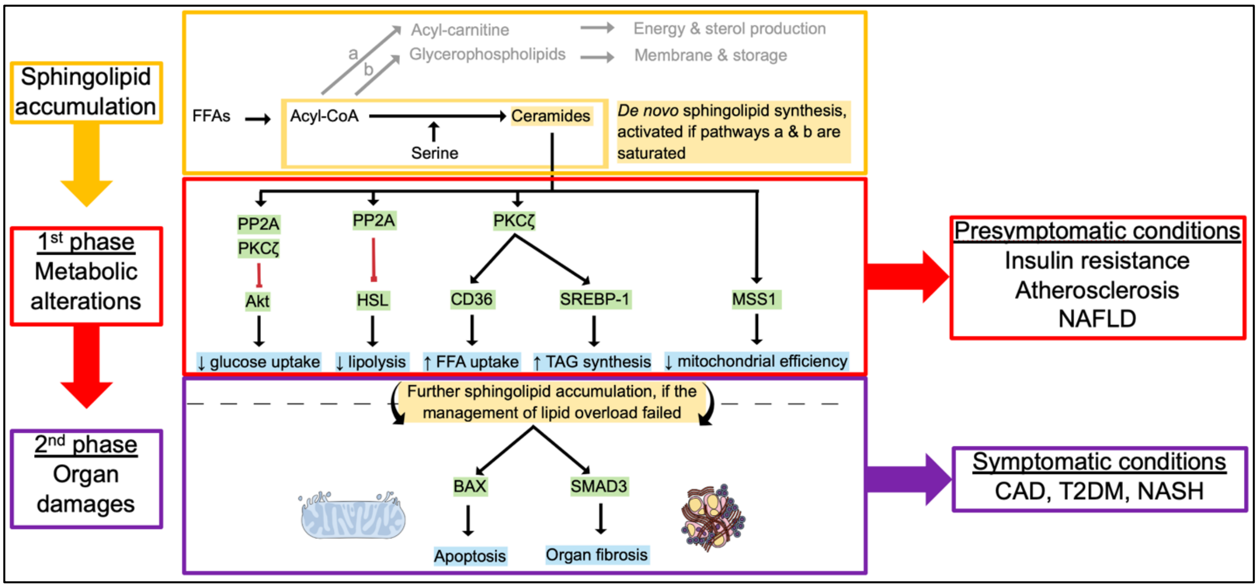

Figure 2: Simplified overview of the molecular alterations and corresponding conditions related to sphingolipid accumulation. Abbreviations: FFA = free fatty acids, acyl-CoA = acyl-coenzyme A, PP2A = protein phosphatase 2 A, PKCζ = protein kinase C zeta, Akt = serine–threonine kinase Akt, HSL = hormone-

sensitive lipase, CD36 = cluster of differentiation 36, also known as a fatty acid translocase, SREBP-1 = sterol regulatory element-binding transcription factor 1, TAG = triacylglycerides, MSS1 = mitochondrial fission factor 1, BAX = bcl-2-like protein 4, SMAD3 = Mothers against decapentaplegic homolog 3, NAFLD = non-alcoholic fatty liver disease, T2DM = type 2 diabetes mellitus, NASH = non-alcoholic steatohepatitis, CAD = coronary artery disease. Blue boxes highlight pathophysiological changes and related clinical conditions. This figure is based on (12, 17).

Sphingolipid profiling – a pathophysiology-based tool for cardiometabolic risk stratification

The profiling of circulating sphingolipids emerged as a powerful tool for assessing the risk of developing cardiometabolic diseases (12). Indeed, they predict the occurrence of CVD and T2DM beyond and independently of current prediction tools (18, 19); and can be non-invasively and reliably detected in blood (12). A machine-learning approach revealed that comprehensive profiling of serum sphingolipids identified patients with CAD independently and more effectively than LDL cholesterol and triglycerides (8). A score combining the blood level of the four most studied sphingolipid species, i.e., ceramide 16:0, ceramide 18:0, ceramide 24:0 and ceramide 24:1, was developed, and it outperformed the 2019 SCORE of the European Society of Cardiology in terms of cardiovascular risk prediction in primary prevention (20, 21, 22). The same score predicted CAD and stroke mortality beyond conventional lipids in primary and secondary prevention (18, 23). Precisely ceramides could also predict T2DM occurrence ten years before the disease was diagnosed (19). This ceramide score has been implemented clinically in private and public hospitals in Finland and at the Cleveland and Mayo Clinics (24, 25). While the utility of sphingolipid phenotyping in cardiometabolic risk stratification is now well-established (15, 16), little is known about the possibilities of lowering sphingolipid levels.

Can exercise reduce circulating sphingolipid levels?

To measure circulating sphingolipids in clinical practice, it is essential to provide patients with evidence-based interventions that reduce sphingolipid levels and quantify the reduction expected from such an intervention. Exercise interventions are ideal candidates for mitigating sphingolipid levels. Indeed, exercise is a powerful polypill to prevent and treat cardiometabolic diseases (26). Exercise interventions are cost-effective, safe, and patient-empowering (27, 28). The molecular pathways underlying the beneficial effects of exercise are increasingly understood (29); however, many mechanisms remain to be elucidated (29, 30). Changes in sphingolipid metabolism might be one of the mechanisms through which exercise optimises cardiometabolic health. Indeed, regular exercise stimulates fatty acid β-oxidation, which redirects lipids toward energy production purposes (pathway a in Figure 2) and could reduce sphingolipid biosynthesis flux (31, 32). In that way, exercise training could reverse pathological sphingolipid accumulation. This hypothesis is supported by the fact that circulating sphingolipids have been reported to be negatively associated with cardiorespiratory fitness (CRF), a potent health marker (33, 34). A preliminary study suggested that regular exercise could mitigate sphingolipid levels (35). However, this study was not powered and examined the sphingolipid response to a 12-week moderate-intensity continuous training. Yet it has been demonstrated that high-intensity interval training (HIIT) is a safe (36, 37) and a more effective way to improve CRF (38, 39, 40) and insulin sensitivity (41, 42, 43, 44) both in healthy individuals and patients with cardiometabolic diseases. Furthermore, this study investigated only a limited number of sphingolipid species (n=7). In contrast, high-throughput targeted lipidomics now allows for the comprehensive analysis of lipid metabolism at the molecular species level (45, 46, 47).

The current research project aims to explore whether and to what extent a CRF-enhancing HIIT-based training programme can lower circulating sphingolipid levels in middle-aged individuals at elevated cardiometabolic risk (50% females). An 'omic-scale sphingolipid profiling will be applied to capture the circulating sphingolipidome comprehensively (48).

2. STUDY OBJECTIVES AND DESIGN

2.1 Hypothesis and primary objective

The current research project aims to determine whether and to what extent a supervised exercise intervention can lower circulating sphingolipid levels in middle-aged females and males at elevated cardiometabolic risk but without symptomatic cardiometabolic disease. Accordingly, the objective is to assess the effect of an 8-week CRF-enhancing HIIT-based training programme on a comprehensive panel of plasma sphingolipid species in sedentary adults aged 40 to 60 years (50% females) with overweight or obesity grade 1 but without any other symptomatic cardiometabolic diseases. The hypothesis is that sphingolipid levels will be reduced following the 8-week HIIT-based training programme.

2.2 Endpoints

The primary endpoints will be changes from pre- to post-intervention levels in the four sphingolipid species included in the ceramide-based score (i.e., ceramide 16:0, ceramide 18:0, ceramide 24:0 and ceramide 24:1) (24, 25).

The secondary endpoints will be changes in the other sphingolipids to be targeted (n=57) in the Homeostatic Model Assessment for Insulin Resistance (HOMA-IR), peak oxygen uptake (VO2peak), flow-mediated dilatation (FMD), retinal vessel analysis, and strain analysis of the heart cavities.

2.3 Study design

This prospective 2-arm, monocentric, randomised controlled trial will include 98 middle-aged participants (50% of females) at elevated cardiometabolic risk. Participants will be randomly allocated to the intervention or control group. It is a clinical trial according to Swiss law (ClinO, Chapter 4) and a risk category A study according to https://www.kofam.ch/de/categoriser/. It will be registered on ClinicalTrials.gov and the Swiss National Clinical Trial Portal. The study will comply with the protocol, the current version of the Declaration of Helsinki, the ICH-GCP or ISO EN 14155 (as far as applicable), and all national legal and regulatory requirements. The study will be conducted at the Department of Sport, Exercise and Health of the University of Basel.

2.4 Study intervention

Screening for eligibility will be performed by phone and consist of the inclusion and exclusion criteria assessment. Participants successfully passing this screening will be invited for a clinical assessment, during which eligibility criteria will be assessed a second time, informed consent will be obtained, a clinical examination will be conducted, and medical clearance be given. Eligible participants will then be invited to a baseline assessment. Instructions for the further procedures of the study will be given.

Group allocation and randomisation

Participants (n= 98, 50% of females) will be randomly allocated, after the baseline examination, to the intervention or the control group. Blocked randomisation will reduce bias and balance allocating participants to both groups (49). The randomisation process will be stratified by age and sex to ensure a balanced allocation. As the randomisation will take place after the baseline examination, participants, exercise scientists, and physicians supervising the intervention will be blinded for group allocation at baseline and not post-intervention.

Training intervention

The exercise intervention will consist of a supervised HIIT (two walking- and one indoor cycling-based session weekly), starting with a habituation week at an intensity of 75% of the maximal heart rate (HRmax). In the following seven weeks, the participants will perform a HIIT based on the following protocol and for a total duration of 45 min per session (modified from Wisløff et al. (50)): warm-up for 10 min at 60%–70% HRmax followed by a high-intensity interval consisting of 4×4 min at 80%–95% HRmax with 3 min of active recovery at 60%–70% HRmax and a 10 min cool-down at 60%–70% HRmax. Heart rate will be monitored during training by Garmin HRM-Dual heart rate sensors combined with Garmin Forerunner 45S watches. Exercise scientists motivate the participants during the intervals and will control each participant's heart rate during and after every training session. This protocol was chosen because it fulfils the requirements of a high-volume HIIT (51), has been extensively studied in both healthy and clinical populations, and its effects on CRF improvement are well-documented (39, 52). In addition, it has been previously used by our research group, with high adherence and absence of drop-out observed in healthy participants and patients with cardiovascular risk factors (53, 54).

Control condition

Asking physically inactive participants to maintain their inactive habits may not reflect realistic conditions and is no longer considered the best option in a randomised controlled exercise intervention (55). Indeed, most participants will be aware of the positive effects of PA - or will become aware of them during an exercise intervention study (55). Further, denying an exercise intervention to participants who would have benefited from it (for instance, participants at risk of cardiometabolic diseases) might be ethically questionable (55). In accordance with current practices (55), control group participants will be informed about the World Health Organization (WHO) PA guidelines at the beginning of the study (56). The control group participants will get a phone call after four weeks to enquire about their well-being.

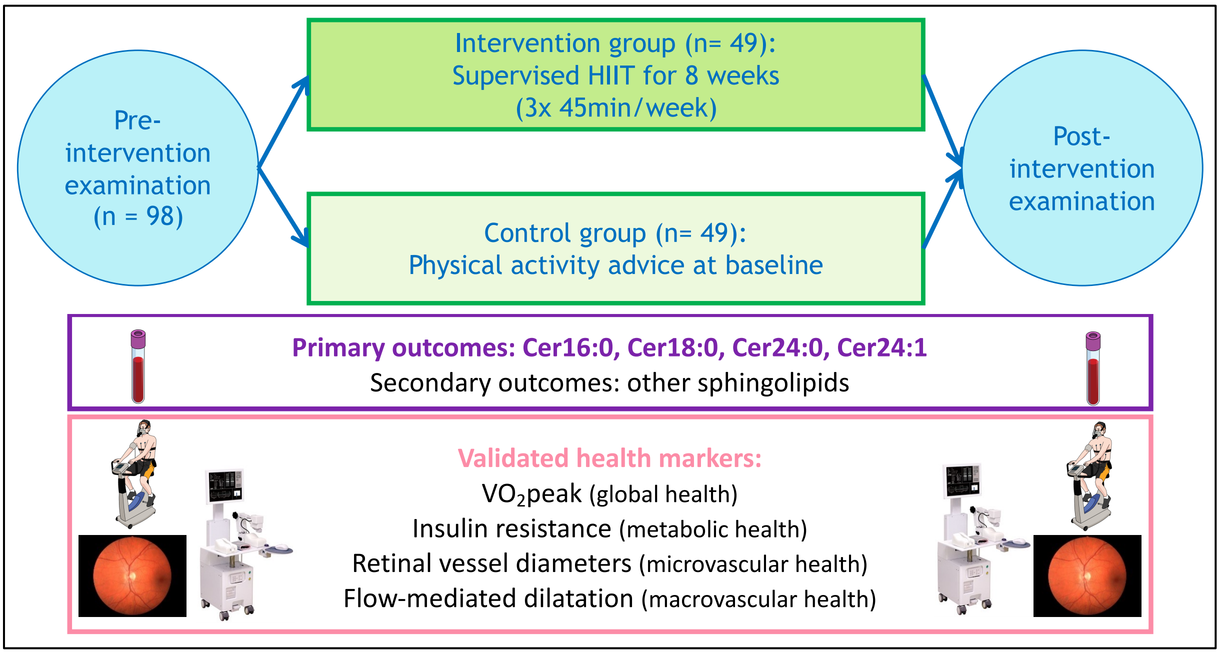

Figure 3: Study design. Abbreviations: HIIT = high intensity interval training, Cer = Ceramide, VO_2peak = peak oxygen uptake

Measurements:

Physical examination and measurements of vital signs (blood pressure, heart rate, respiration rate, oxygen saturation and body temperature), a resting and exercise ECG will be part of the clinical assessment to screen for cardiac contraindications to maximal exertion. Habitual PA will be assessed using the Global Physical Activity Questionnaire (57), which has been validated in a Swiss context (58). Maximal cardiopulmonary exercise testing (CPET) will be conducted on a cycle ergometer to determine VO_2peak, peak heart rate and power output (Ergoselect 200; Ergoline, Bitz, Germany). After the training programme, a second CPET will be performed to verify that the exercise intervention effectively improved CRF.

Height and body weight will be measured to the nearest 0.5 cm and 0.1 kg, respectively, and the body mass index will be calculated. Body composition will be analysed before and after the intervention by the gold-standard, dual-energy x-ray absorptiometry (DXA) using a GE Lunar iDXA® machine (GE Lunar Inc., Madison, WI; software version 13.10). Study participants will fast within 6 h prior to the measurement. Absolute and relative measures of total body fat mass and fat-free mass will be obtained. During the measurement, patients will be in a supine position (59). The effective radiation dose from a single whole-body DXA (< 10 µSv) is similar to the normal background radiation received over one day at sea level (60). Consequently, the effective radiation dose per study participant in the present study will be < 20 µSv, which is similar to the normal background radiation received over two days at sea level. For comparison purposes, body composition will also be analysed before and after the intervention by four-segment bioelectrical impedance analysis using the InBody 720 (Inbody Co. Ltd., Seoul, South Korea). Before the measure, participants must refrain from intense PA for 24 h, fast for at least two hours, and be asked to void their bladder.

Trained medical staff will draw blood samples by venepuncture of the cubital fossa following an overnight fast pre-and post-intervention. The total volume of blood samples will be ca. 40 mL (2 × 2.7 mL potassium-EDTA, 2 × 7.5 mL serum-monovette, and 1 × 7.5 mL Li-Heparin). Blood samples will be immediately centrifuged, and the serum aliquots will be frozen at − 80 °C. Planned blood analyses for essential characterisation of risk factor profiles include total cholesterol, LDL- and HDL-cholesterol, triglycerides, and HbA1c. Glucose and insulin will also be measured to estimate insulin resistance using the Homeostatic Model Assessment for Insulin Resistance (HOMA-IR) (61). A high-coverage method using reversed-phase liquid chromatography coupled to tandem mass spectrometry (RPLC-MS/MS) will be applied to quantify an extensive panel of circulating sphingolipids (n=61) (48).

To our knowledge, no evidence exists that food intake influences sphingolipid levels. Nevertheless, each participant will receive standardised, pre-packaged meals for the two days preceding blood sampling to minimise potential confounding. All participants will be fed to energy balance. Energy requirements will be calculated with the formulas of Mifflin St. Joer (62) and the National Institute of Diabetes and Digestive and Kidney Diseases Body Weight Planner (63, 64). All diets will contain ~55% energy from carbohydrates, ~25% from fat, and ~20% from protein. To monitor diet adherence, participants will be instructed to return all non-consumed foods from the pre-packaged meals to the lab and take photos of additionally consumed foods for later analysis. Participants will also be asked to refrain from alcohol consumption during the dietary control period (i.e., the two days preceding blood sampling). For the rest of the study, participants will be advised to eat as usual.

Retinal vessel diameters, a novel surrogate of microvascular health that responds positively to exercise intervention, will be assessed pre-and post-intervention as described in detail by Hanssen et al. (65). Arteriolar and venular diameters will be measured using semiautomatic software based

on a modified fundus camera (450FF; Carl Zeiss, Jena, Germany). Three valid images of the right eye retina will be taken at an angle of 45° and with the optic disc in the centre. Retinal arterioles and venules within a distance of 0.5 to 1 disc diameter from the optic disc will be identified semi-automatically (Vesselmap 2; IMEDOS Systems, Jena, Germany). Vessel diameters will be averaged to central retinal arteriolar (CRAE) and venular equivalents (CRVE), as well as their ratio, using the Parr–Hubbard formula described elsewhere (66). This reliable method has inter- and intra-observer interclass correlation coefficients between 0.78 to 0.99 (66, 67).

Brachial artery flow-mediated dilatation (FMD) reflects endothelial function as an early marker of atherosclerotic arterial damage (68). It provides an in vivo, noninvasive, direct measure of artery function and health (69). There is increasing evidence that FMD in humans is associated with traditional risk factors, cardiovascular diseases, and heart failure and predicts cardiovascular events and all-cause mortality (68, 70). It is sensitive to changes in behavioural health shown for the Mediterranean diet, acute mental stress or exercise training (71, 72, 73). Measurement of FMD of the brachial artery follows after at least 15-20min of rest in the supine position using a high-resolution linear ultrasound array transducer with a semi-automatic detection program of the intima-media complex (EF, Unex Corporation, Nagoya, Japan). An occlusion cuff will be wrapped around the forearm with the proximal edge of the cuff at the elbow. Longitudinal images of the right brachial artery (typically at 3-5 cm above the elbow) are recorded at baseline and after cuff deflation following supra systolic compression (50 mmHg over the systolic blood pressure value) of the right forearm for 5 minutes until 3 minutes after deflation. Inter-reader and inter-session reliability of the FMD measurement were acceptable (74, 75).

Echocardiographic assessment, particularly atrial strain analysis—an emerging ultrasound-based technique—facilitates the comprehensive evaluation of atrial mechanics, offering invaluable insights into atrial fibrosis (76). Atrial fibrosis, in turn, is a hallmark of atrial structural remodelling, which leads to atrial fibrillation, the most common cardiac arrhythmia with an ever-increasing prevalence (77, 78). Strain analysis, a highly sensitive measure of cardiac function, has demonstrated responsiveness to even short periods of endurance exercise training, as observed in a 4-week HIIT intervention (79).
Standardised echocardiographic images will be systematically acquired in the supine position, adhering to international standards (80, 81). The imaging process will use a state-of-the-art ultrasound system (Philips Epiq 7 Ultrasound System, Koninklijke Philips N.V., Amsterdam) equipped with a compatible broadband sector array transducer (S5-1, Philips, Koninklijke Philips N.V., Amsterdam). Post-acquisition, echocardiographic images will undergo meticulous analysis for strain parameters within the ultrasound workspace, utilising advanced software (TOMTEC Imaging Systems GmbH, Unterschleissheim, Germany).

3. STUDY POPULATION AND STUDY PROCEDURES

3.1 Inclusion and exclusion criteria, justification of study population

This randomised controlled trial will include 98 participants (50% of females) aged between 40 and 60 years at elevated cardiometabolic risk. They will be randomised to the intervention and control groups. To investigate whether circulating sphingolipid levels can be reduced following an 8-week HIIT intervention, it is ideal to include patients at elevated cardiometabolic risk but without any symptomatic cardiometabolic diseases. Indeed, these patients are likely situated in the first phase of sphingolipid accumulation, which is believed to be reversible.

Inclusion criteria:

Participants fulfilling all the following inclusion criteria are eligible for the study:

- female or male sex,
- aged between 40 and 60 years,
- body mass index between 25.0 and 34.9 kg/m^2,
- sedentary lifestyle, defined as not meeting the WHO guidelines on PA, i.e., at least 150 minutes of moderate-intensity aerobic PA per week as well as muscle-strengthening activities on two or more days per week (28),
- medical clearance for HIIT by a study physician (including vital sign evaluation, clinical examination, resting and exercise ECG),
- informed consent as documented by signature.

Exclusion criteria:

Participants fulfilling one of the following exclusion criteria will not be included in the study:

- known pregnancy or breastfeeding,
- any current exercise-limiting musculoskeletal conditions of the lower limbs,
- any known current or chronic conditions limiting exhaustive exercise,
- known diabetes mellitus of any type,
- dyslipidaemia, if pharmaceutically treated,
- arterial hypertension ≥160/100 mmHg, pharmaceutically treated or not,
- any other known cardiovascular disease,
- known NASH,
- known macular degeneration, glaucoma, or high intraocular pressure (≥20 mm Hg),
- particular diet (vegetarian, vegan, lactose-free, gluten-free, or FODMAP-low diet (fermentable oligosaccharides, disaccharides, monosaccharides, and polyols),
- intake of glucagon-like peptide-1 analogues, orlistat or any weight-loss drug,
- inability to follow the procedures of the study, e.g., due to linguistic or cognitive problems,
- concomitant involvement in another interventional trial or participation in another interventional trial in the last four weeks.

3.2 Recruitment, screening, and informed consent procedure

Inhabitants from the Basel area will be invited to participate in this study via advertisements in local newspapers and on diverse social media channels. A similar recruitment process was successful for the EXAMIN AGE and HyperVasc studies (54, 82). Suitable patients of the Exercise Medicine Clinic of the DSBG will also be offered to participate.

The investigators will explain during a phone call the nature of the study, its purpose, the procedures involved, the expected duration, the potential risks and benefits, and any discomfort it may entail to each potential participant. The inclusion and exclusion criteria will be explained to each potential participant. Each participant will be informed that participation in the study is voluntary and that they may withdraw from the study at any time without reason.

Following the phone call, all potential participants will be provided via e-mail with a participant information sheet and a consent form describing the study and providing sufficient information for participants to make an informed decision about their participation in the study. Participants will be given at least 24h (after receiving the participant information sheet and consent form) to decide whether to participate. The recruitment will be continuous until the targeted 98 participants are enrolled. The formal consent of a participant, using the approved consent form, will be obtained before the participant is submitted to any study procedure. The study participant will receive a copy of the signed informed consent. The consent form will be retained as part of the study records.

The study will be conducted in accordance with the Declaration of Helsinki.

Potential participants will be screened for initial eligibility in a first personal visit at the Department of Sport, Exercise and Health of the University of Basel, which will take place after potential participants return the signed consent form. Inclusion and exclusion criteria will be carefully reviewed during the first personal visit by asking the questions in the document "Fragen Screening" point-by-point. A clinical assessment (including vital sign evaluation, clinical examination, height and weight measurement, body mass index (BMI) calculation, resting and exercise ECG) and medical clearance will also take place during this first visit. Eligible participants will then be invited to a baseline assessment.

3.3 Study procedures

First contact by phone – study information:
The investigators will telephone each potential participant to explain the nature of the study, its purpose, the procedures involved, the expected duration, the potential risks and benefits, and any discomfort it may entail. The inclusion and exclusion criteria will be explained to each potential participant. Each participant will be informed that participation in the study is voluntary and that they may withdraw from the study at any time without reason.

1st visit – clinical and eligibility assessment:
A clinical assessment which includes vital sign evaluation (blood pressure, heart rate, respiration rate, oxygen saturation, and body temperature), physical examination, height and weight measurement, and BMI calculation, will be conducted. Habitual PA will be assessed using the Global Physical Activity Questionnaire (57, 58).
A resting and exercise ECG will be realised to screen for cardiac contraindications to maximal exertion. Maximal cardiopulmonary exercise testing (CPET) will be conducted on a cycle ergometer to determine VO_2peak, peak heart rate and power output (Ergoselect 200; Ergoline, Bitz, Germany). Finally, medical clearance will be given during this first visit.
The screening for eligibility will consist of the inclusion and exclusion criteria assessment (see section 3.1 for inclusion and exclusion criteria). Participants successfully passing this screening will be invited for a baseline assessment. Instructions for the further procedures of the study will be given.

2nd visit – collection of standardised meals
Each participant will collect, at least three days before the baseline assessment, standardised, prepackaged meals for the two days preceding blood sampling to minimise potential confounding. Depending on the delay between the first visit and the baseline assessment, this second visit can be combined with the first visit.

3rd visit – baseline assessment
Body composition will be analysed before and after the intervention by DXA and bioelectrical impedance analysis. Trained medical staff will draw blood samples by venepuncture of the cubital fossa following an overnight fast pre-and post-intervention. Retinal vessel diameters, a novel surrogate of microvascular health which responds positively to exercise intervention, will be assessed pre-and post-intervention as extensively described by Hanssen et al. (65). Brachial artery flow-mediated dilatation (FMD) reflects endothelial function as an early marker of atherosclerotic arterial damage (68). It provides an in vivo, noninvasive, direct measure of artery function and health (69). Echocardiographic strain analysis of the atria offers insights into cardiac mechanical adaptation in response to elevated sphingolipid levels (83).

Intervention (8-week HIIT vs. PA recommandations)

The exercise intervention will consist of a supervised HIIT (two walking- and one indoor cycling-based session weekly), starting with a habituation week at an intensity of 75% of the maximal heart rate (HRmax). In the following seven weeks, the participants will perform a HIIT based on the following protocol and for a total duration of 45 min per session (modified from Wisløff et al. (50)): warm-up for 10 min at 60%–70% HRmax followed by a high-intensity interval consisting of 4×4 min at 80%–95% HRmax with 3 min of active recovery at 60%–70% HRmax and a 10 min cool-down at 60%–70% HRmax. Heart rate will be monitored during training by Garmin HRM-Dual heart rate sensors combined with Garmin Forerunner 45S watches. Exercise scientists motivate the participants during the intervals and will control each participant's heart rate during and after every training session. This protocol was chosen because it fulfils the requirements of a high-volume HIIT (51), has been extensively studied in both healthy and clinical populations, and its effects on CRF improvement are well-documented (39, 52). In addition, it has been previously used by our research group, with high adherence and absence of drop-out observed in healthy participants and patients with cardiovascular risk factors (53, 54). The control group will get PA recommendations based on current guidelines (56). The control group participants will get a phone call after four weeks to enquire about their well-being.

4th visit – collection of standardised meals

Each participant will collect, at least three days before the baseline assessment, standardised, pre-packaged meals for the two days preceding blood sampling at post-intervention assessment to minimise potential confounding. For the intervention group, this visit can be combined with a training session.

5th visit – post-intervention assessment

After completing the 8-week intervention, the same measurements from the baseline assessment will be repeated (including vital sign evaluation, clinical examination, height and weight measurement, BMI calculation, resting and exercise ECG). A second CPET will be performed to verify that the exercise intervention effectively improved CRF. Habitual PA will be re-assessed using the Global Physical Activity Questionnaire (57, 58).

All blood samples will be stored at -80°C at the Department of Sport, Exercise and Health of the University of Basel before being delivered to the Metabolomics Unit of the Faculty of Biology and Medicine at the University of Lausanne. A high-coverage method using reversed-phase liquid chromatography coupled to tandem mass spectrometry (RPLC-MS/MS) will be applied to quantify the 61 circulating sphingolipids (48).

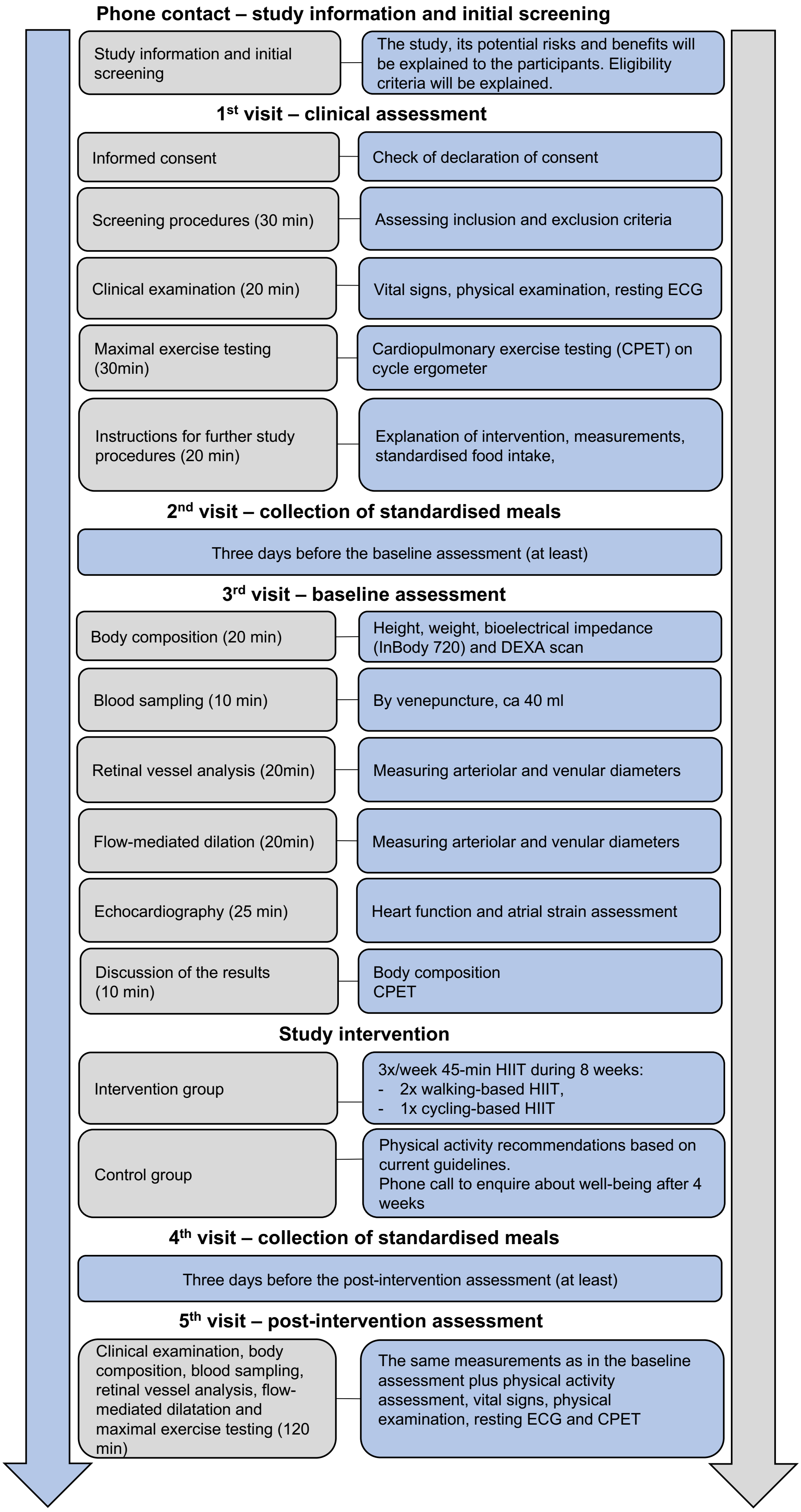
Phone contact – study information and initial screening
Study information and initial screening
The study, its potential risks and benefits will be explained to the participants. Eligibility criteria will be explained.
1st visit – clinical assessment
Informed consent
Check of declaration of consent
Screening procedures (30 min)
Assessing inclusion and exclusion criteria
Clinical examination (20 min)
Vital signs, physical examination, resting ECG
Maximal exercise testing (30min)
Cardiopulmonary exercise testing (CPET) on cycle ergometer
Instructions for further study procedures (20 min)
Explanation of intervention, measurements, standardised food intake,
2nd visit – collection of standardised meals
Three days before the baseline assessment (at least)
3rd visit – baseline assessment
Body composition (20 min)
Height, weight, bioelectrical impedance (InBody 720) and DEXA scan
Blood sampling (10 min)
By venepuncture, ca 40 ml
Retinal vessel analysis (20min)
Measuring arteriolar and venular diameters
Flow-mediated dilation (20min)
Measuring arteriolar and venular diameters
Echocardiography (25 min)
Heart function and atrial strain assessment
Discussion of the results (10 min)
Body composition
CPET
Study intervention
Intervention group
3x/week 45-min HIIT during 8 weeks:
- 2x walking-based HIIT,
- 1x cycling-based HIIT
Control group
Physical activity recommendations based on current guidelines.
Phone call to enquire about well-being after 4 weeks
4th visit – collection of standardised meals
Three days before the post-intervention assessment (at least)
5th visit – post-intervention assessment
Clinical examination, body composition, blood sampling, retinal vessel analysis, flow-mediated dilatation and maximal exercise testing (120 min)
The same measurements as in the baseline assessment plus physical activity assessment, vital signs, physical examination, resting ECG and CPET

Figure 4: Schedule and sequence of study procedures as planned for study visits. Abbreviations: ECG = electrocardiogram, CPET = cardio-pulmonary exercise testing, HIIT = high intensity interval training

3.4 Withdrawal and discontinuation

Every participant enrolled in the study will always be able to withdraw his participation for further assessments without any reason. Already collected data will remain available for data analysis. Participants will be removed from the study if exclusion criteria are encountered during the study or if participants withdraw informed consent.

4. STATISTICS AND METHODOLOGY

4.1 Statistical analysis plan and sample size calculation

The sample size calculation and statistical analysis plan of the SphingoFIT study has been developed in close collaboration with Dr Denis Infanger, advising statistician at the DSBG. The sample size calculation is based on the four primary endpoints, which are also the species entering the ceramide score (i.e., ceramide 16:0, ceramide 18:0, ceramide 24:0 and ceramide 24:1) (24, 25). It was hypothesised that 1) log2-transformed pre-intervention sphingolipid levels are similar in both the intervention and the control groups and 2) the standard deviation of log2-transformed sphingolipid levels correspond to 0.475 (which is the average standard deviation obtained for the four sphingolipids mentioned above in participants of the COmPLETE Health study aged 40-60 years (84)). As the results of the SphingoHIIT study are not available yet, the effect size was estimated based on the unique intervention study available in the literature (35). The authors of this study reported an average effect size (expressed as a geometric mean ratio) of 1.17. This means that the geometric mean of the sphingolipid levels was 1.17 times higher in control than in the intervention group following the exercise intervention. Assuming a realistic correlation coefficient ρ of 0.6 between pre-and post-intervention values for sphingolipid levels, an analysis of covariance (ANCOVA) was used to calculate the sample size. A result of 49 participants per group for a power of 80% was obtained (Figure 4). Notably, a drop-out rate of 10% was considered in the sample size calculation (55).

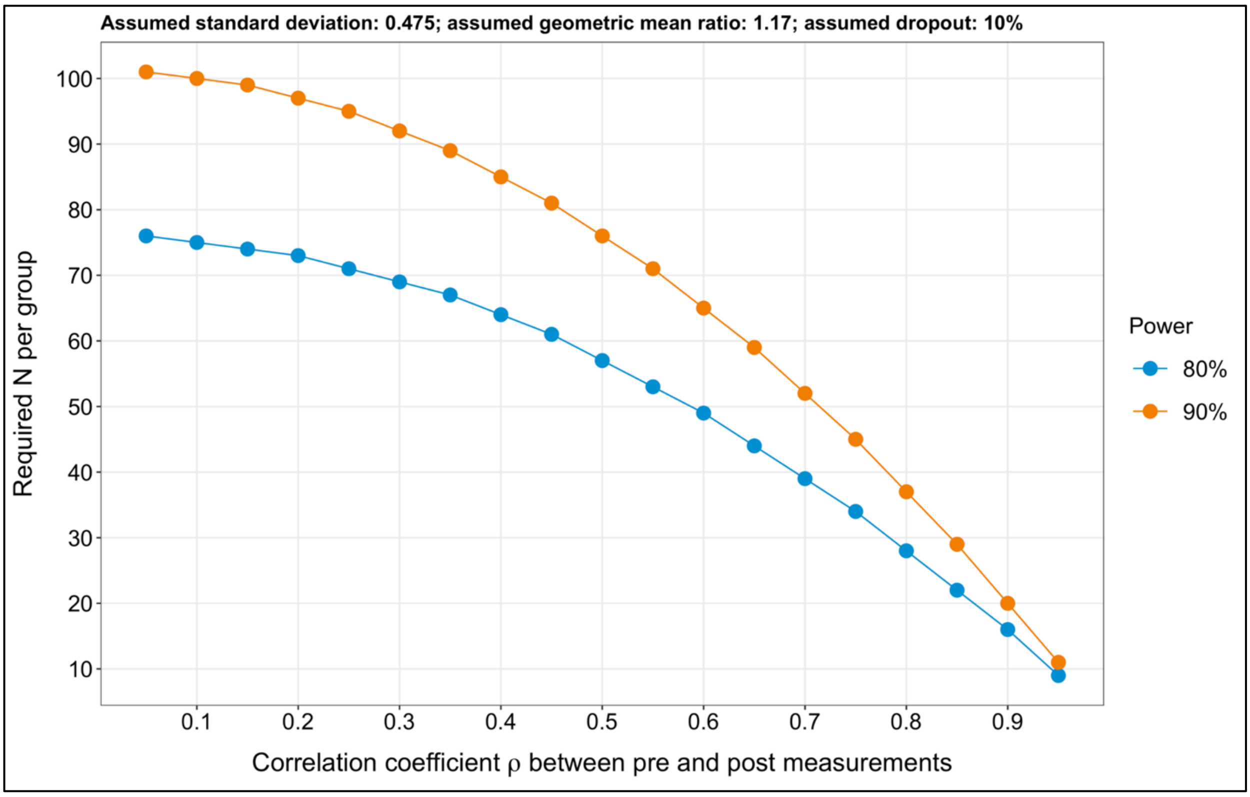

Figure 5: Sample size calculation

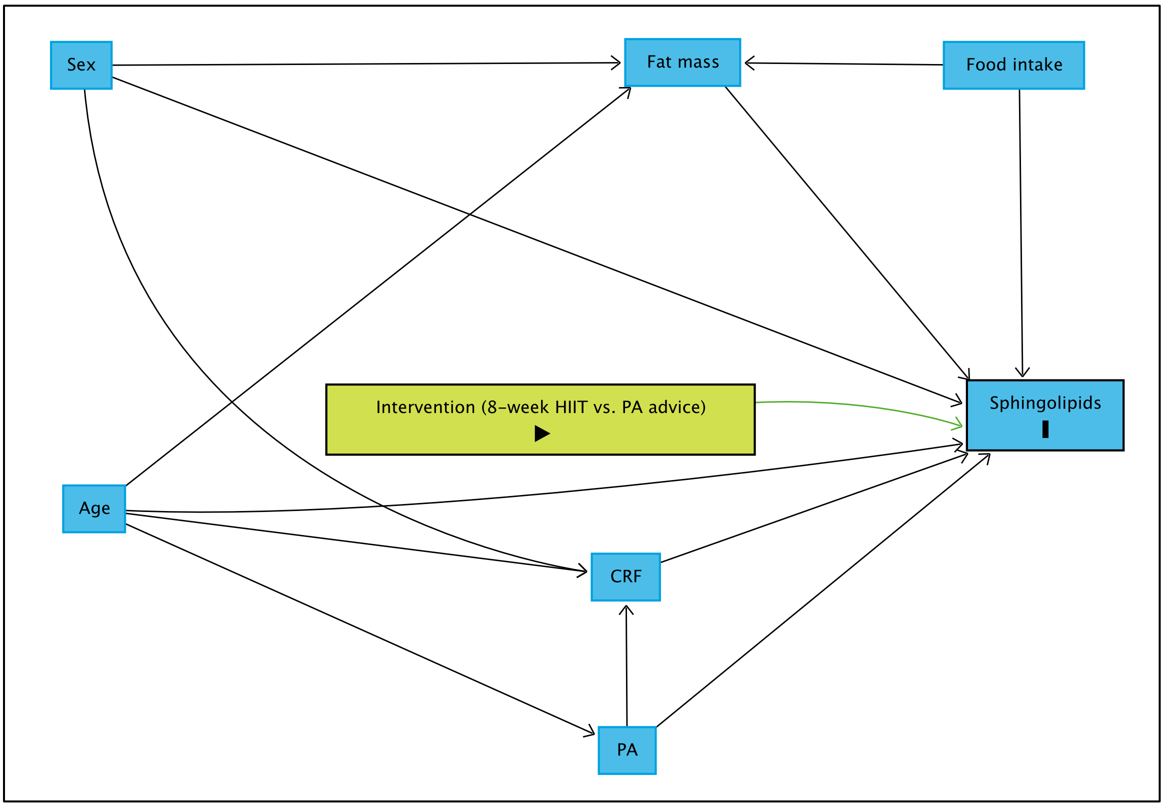

Figure 6: Directed acyclic graph representing the effect of HIIT on sphingolipid levels and other influencing variables. Abbreviations: HIIT = high-intensity interval training, CRF = cardiorespiratory fitness, PA = physical activity, SL = sphingolipid levels.

Statistical analyses:
An ANCOVA will be run for each sphingolipid species to estimate the effect of the intervention (85). The post-intervention sphingolipid value will be the dependent variable. In contrast, the pre-intervention sphingolipid value, a group variable (i.e., intervention or control group) and all control variables will be the independent variables. To identify control variables to be included in the models, we drew a causal-directed acyclic graph (DAG) using DAGitty (86). Age, sex, body fat mass and CRF were identified as variables to be included in the models to reduce the outcome variation and improve the precision of the average causal effect of the intervention (87). The design will control food intake (as each participant will receive individualised, pre-packaged meals for the two preceding blood sampling) and PA. Graphical methods will be used to assess the normal distribution of data. If the data are not normally distributed, sphingolipid concentrations will be log-transformed. The Benjamini-Hochberg method will adjust P-values for multiple testing (88). The significance level is set at $\alpha = 0.05$; all tests will be two-sided. All analyses will be done according to the intention-to-treat principle. Statistical analyses will be conducted using R (version 4.0.2 or later). The study will report its results in compliance with the CONSORT statement (89).

4.2 Handling of missing data and drop-outs

A complete case analysis will be done if the proportion of missing data is below 5%. Otherwise, we will consider multiple imputations. A drop-out rate of 10% was considered in the sample size calculation.

5. REGULATORY ASPECTS AND SAFETY

5.1 Local regulations / Declaration of Helsinki

This study will be conducted in compliance with the protocol, the current version of the Declaration of Helsinki, the ICH-GCP, the HRA, as well as other locally relevant legal and regulatory requirements.

5.2 (Serious) Adverse Events and notification of safety and protective measures

An Adverse Event (AE) is any untoward medical occurrence in a study participant which does not necessarily have a causal relationship with the trial procedure. An AE can therefore be any unfavourable or unintended finding, symptom, or disease temporally associated with a trial procedure, whether related to it or not.

A Serious Adverse Event (SAE) (ClinO, Art. 63) is any untoward medical occurrence that:
- Results in death or is life-threatening,
- Requires in-patient hospitalisation or prolongation of existing hospitalisation, or
- Results in persistent or significant disability or incapacity

The investigator and Sponsor-Investigator make a causality assessment of the event to the trial intervention (see table below based on the terms given in ICH E2A guidelines). Any event assessed as possibly, probably or related is classified as related to the trial intervention.

Relationship	Description

Definitely	Temporal relationship<br>Improvement after dechallenge*<br>Recurrence after rechallenge<br>(or other proof of drug cause)
Probably	Temporal relationship<br>Improvement after dechallenge<br>No other cause evident
Possibly	Temporal relationship<br>Other cause possible
Unlikely	Any assessable reaction that does not fulfil the above conditions
Not related	Causal relationship can be ruled out
*Improvement after dechallenge only taken into consideration, if applicable to reaction	

Both Investigator and Sponsor-Investigator make a severity assessment of the event as mild, moderate, or severe. Mild means the complication is tolerable, moderate means it interferes with daily activities and severe means it renders daily activities impossible.

Reporting of SAEs (see ClinO, Art. 63)

All SAEs are documented and reported immediately (within a maximum of 24 hours) to the Sponsor and to the Investigator of the study.
If it cannot be excluded that the SAE occurring in Switzerland is attributable to the intervention under investigation, the Investigator reports it to the Ethics Committee via BASEC within 15 days.

Follow-up of (Serious) Adverse Events

Participants suffering from adverse events are treated medically, and an appropriate follow-up is ensured when the participant fully recovers. The follow-up process is documented and submitted to the Ethics Committee.

Notification of safety and protective measures (see ClinO, Art 62, b)

If immediate safety and protective measures have to be taken during the study, the investigator notifies the Ethics committee of these measures and the circumstances necessitating them within seven days.

5.3 (Periodic) safety reporting

An annual safety report (ASR) is submitted once a year to the local Ethics Committee by the Investigator (ClinO, Art. 43 Abs).

5.4 Radiation

The effective radiation dose from a single whole-body DXA (< 10 µSv) is similar to the normal background radiation received over one day at sea level (60). Consequently, the effective radiation dose per study participant in the present study will be < 20 µSv, which is similar to the normal background radiation received over two days at sea level.

5.5 Pregnancy

Not applicable

5.6 Amendments

Substantial changes to the study setup and organisation, the protocol and relevant study documents are submitted to the Ethics Committee for approval before implementation. Under emergency circumstances, deviations from the protocol to protect the rights, safety and well-being of human subjects may proceed without prior approval of the Ethics Committee. Such deviations shall be documented and reported to the Ethics Committee as soon as possible. A list of all non-substantial amendments will be submitted once a year to the competent EC together with the ASR.

5.7 Notification and reporting upon completion, discontinuation or interruption of the study

Upon regular study completion, the Ethics Committee is notified via BASEC within 90 days (ClinO, Art. 38).

The Sponsor-Investigator may terminate the study prematurely according to certain circumstances, e.g.

- Ethical concerns,
- Insufficient participant recruitment,
- When the safety of the participants is doubtful or at risk (e.g. when the benefit-risk assessment is no longer positive),
- Alterations in accepted clinical practice that make the continuation of the study unwise, or
- Early evidence of harm or benefit of the experimental intervention.

Upon premature study termination or study interruption, the Ethics Committee is notified via BASEC within 15 days (ClinO, Art. 38).

A final report is submitted to the Ethics Committee via BASEC within a year after completion or discontinuation of the study, unless a longer period is specified in the protocol (ClinO, Art. 38).

5.8 Insurance

The study is risk category A according to the Kofam risk assessment. In the event of study-related damage or injuries, the liability of the University of Basel provides compensation, except for claims that arise from misconduct or gross negligence.

6. FURTHER ASPECTS

6.1 Overall ethical considerations

Exercise as a prevention method for cardiometabolic diseases is a safe and broadly accessible approach. This study will be another step in generating scientific evidence regarding HIIT training and its influence on the sphingolipidome, leading to new preventive and therapeutic strategies in treating cardiometabolic disease. Positive findings from the study can help reduce the gap in health care between low-income and high-income countries, as HIIT training is feasible in low-income countries due to its practicality and low cost.

6.2 Risk-benefit assessment

The SphingoFIT study is designed so the participants can participate in an 8-week training program for free. HIIT brings the risk of short-term exhaustion or muscle soreness. Another posed risk is suffering from a musculoskeletal injury through training. These risks are minimised by dividing the training into a walking- and cycling-based part, thus alternating load and minimising the load on the joints. The risk for cardiovascular events through exercise is reduced by performing a resting and an exercise ECG before starting the intervention to screen for pathological changes in the heart. The potential benefit of gaining fitness and thus improving different health parameters outweigh, however, these risks. All subjects also benefit from receiving medical status information and expensive and complex examinations that can give them detailed information about their fitness status. In addition, their participation helps generate knowledge, forming the basis for new prevention methods.
All involved parties will keep the participant's data strictly confidential. Data will be entered in protected files on the server of the Department of Sport, Exercise and Health of the University of Basel. Access to these files will be restricted to study group members who agreed on confidentiality.

7. QUALITY CONTROL AND DATA PROTECTION

7.1 Quality measures

The sponsor, the Ethics Committee, or an independent trial monitor may visit the research site anytime for quality assurance. Direct access to the source data and all study-related files will be granted on such occasions. All involved parties will keep the participant's data strictly confidential. Data will be entered in protected files on the server of the Department of Sport, Exercise and Health of the University of Basel. Access to these files will be restricted to study group members who agreed on confidentiality. Trained physicians will perform baseline assessments that received standardised instructions concerning the study procedures and completed a good clinical practice course. The study intervention will be supervised by personnel trained in all essential study aspects to standardise processes.

7.2 Data recording and source data

A CRF (Case Report Form) will be created for each participant in which the participant's data is only stored in encrypted form. No conclusions about personal data such as name, date of birth or telephone number can be drawn from this. Indeed, each enrolled participant will be pseudonymised by a unique subject identifier (participant ID).

7.3 Confidentiality and coding

Trial and participant data will be handled with uttermost discretion and is only accessible to authorised personnel who require the data to fulfil their duties within the scope of the study. On the CRFs and other examination-specific documents, participants will only be identified by a unique participant number. The participant identification list will only be stored on the server of the Department of Sport, Exercise and Health of the University of Basel. Access to these documents will be restricted to the Principal Investigator and selected study personnel if contact with participants is required.
In the SphingoFIT study, only coded non-genetic data will be used. Biological material in this study will not be identified by participant name but by a unique participant number. Biological

material will be appropriately stored in a restricted area only accessible to authorised personnel. To analyse the circulating sphingolipids using RPLC-MS/MS, probes will be sent to:

Metabolomics Platform
Faculty of Biology and Medicine
Quartier UNIL-CHUV
Rue du Bugnon 19
CH-1005

Dr Julijana Ivanisevic, head of the Metabolomics Platform, will receive the probes. She will guarantee confidentiality during the whole analysis duration when handling the investigations and the resulting data.

7.4 Retention and destruction of study data and biological material

All study data will be archived locally in a password-protected file on a local server for ten years after study termination or premature termination of the study at the Department of Sport, Exercise and Health of the University of Basel. Biological material collected during the SphingoFIT study will be stored at the Department of Sport, Exercise and Health for ten years after study termination. Biological material will be eliminated after these ten years, and the destruction of this material will be documented using a confirmation sheet, which will be stored locally on a computer at the Department of Sport, Exercise and Health of the University of Basel.

8. FUNDING / PUBLICATION / DECLARATION OF INTEREST

The principal investigator declares that there is no current conflict of interest. The Research Fund for Excellent Junior Researchers of the University of Basel granted Dr Justin Carrard an 80,000 CHF grant to pursue this study. This will cover a part of Justin Carrard's position and 4,040 CHF of consumables. The Swiss Life Jubilee Foundation granted Dr Justin Carrard with a 15,000 CHF grant to cover further consumables and laboratory analysis. Further grant applications are pending. In case they are unsuccessful, Prof Arno Schmidt-Trucksäss will cover the resting costs. The SphingoFIT study will be registered on clinicaltrials.gov, and results generated from this study will be published in a peer-reviewed journal and held as a conference presentation.

9. REFERENCES

1. Lozano R, Naghavi M, Foreman K, Lim S, Shibuya K, Aboyans V, et al. Global and regional mortality from 235 causes of death for 20 age groups in 1990 and 2010: a systematic analysis for the Global Burden of Disease Study 2010. The Lancet. 2012;380(9859):2095-128.
2. Global, regional, and national disability-adjusted life years (DALYs) for diseases and injuries and healthy life expectancy (HALE), 1990 to 2019: quantifying the epidemiological transition. [Internet]. Seattle, WA: Institute for Health Metrics and Evaluation, University of Washington. [cited 13.07.2021]. Available from: http://vizhub.healthdata.org/gbd-compare.
3. Hunter DJ, Reddy KS. Noncommunicable Diseases. New England Journal of Medicine. 2013;369(14):1336-43.
4. Benziger CP, Roth GA, Moran AE. The Global Burden of Disease Study and the Preventable Burden of NCD. Glob Heart. 2016;11(4):393-7.
5. Verma KP, Inouye M, Meikle PJ, Nicholls SJ, Carrington MJ, Marwick TH. New Cardiovascular Risk Assessment Techniques for Primary Prevention: JACC Review Topic of the Week. Journal of the American College of Cardiology. 2022;80(4):373-87.
6. Sachdeva A, Cannon CP, Deedwania PC, Labresh KA, Smith SC, Jr., Dai D, et al. Lipid levels in patients hospitalized with coronary artery disease: an analysis of 136,905 hospitalizations in Get With The Guidelines. Am Heart J. 2009;157(1):111-7.e2.
7. Martins AMA, Paiva MUB, Paiva DVN, de Oliveira RM, Machado HL, Alves LJSR, et al. Innovative Approaches to Assess Intermediate Cardiovascular Risk Subjects: A Review From Clinical to Metabolomics Strategies. Frontiers in Cardiovascular Medicine. 2021;8.
8. Poss AM, Maschek JA, Cox JE, Hauner BJ, Hopkins PN, Hunt SC, et al. Machine learning reveals serum sphingolipids as cholesterol-independent biomarkers of coronary artery disease. The Journal of Clinical Investigation. 2020;130(3):1363-76.
9. Berkowitz L, Cabrera-Reyes F, Salazar C, Ryff CD, Coe C, Rigotti A. Sphingolipid Profiling: A Promising Tool for Stratifying the Metabolic Syndrome-Associated Risk. Frontiers in Cardiovascular Medicine. 2022;8.
10. Hannun YA, Obeid LM. Sphingolipids and their metabolism in physiology and disease. Nat Rev Mol Cell Biol. 2018;19(3):175-91.
11. Poss AM, Summers SA. Too Much of a Good Thing? An Evolutionary Theory to Explain the Role of Ceramides in NAFLD. Frontiers in Endocrinology. 2020;11.
12. Nicholson RJ, Norris MK, Poss AM, Holland WL, Summers SA. The Lard Works in Mysterious Ways: Ceramides in Nutrition-Linked Chronic Disease. Annual Review of Nutrition. 2022;42(1):115-44.
13. Chaurasia B, Summers SA. Ceramides – Lipotoxic Inducers of Metabolic Disorders. Trends Endocrinol Metab. 2015;26(10):538-50.
14. Öörni K, Jauhiainen M, Kovanen PT. Why and how increased plasma ceramides predict future cardiovascular events? Atherosclerosis. 2020;314:71-3.
15. Choi RH, Tatum SM, Symons JD, Summers SA, Holland WL. Ceramides and other sphingolipids as drivers of cardiovascular disease. Nat Rev Cardiol. 2021;18(10):701-11.
16. Tippetts TS, Holland WL, Summers SA. Cholesterol - the devil you know; ceramide - the devil you don't. Trends Pharmacol Sci. 2021;42(12):1082-95.
17. Summers SA. Editorial: The Role of Ceramides in Diabetes and Cardiovascular Disease. Frontiers in Endocrinology. 2021;12.
18. Laaksonen R, Ekroos K, Sysi-Aho M, Hilvo M, Vihervaara T, Kauhanen D, et al. Plasma ceramides predict cardiovascular death in patients with stable coronary artery disease and acute coronary syndromes beyond LDL-cholesterol. European heart journal. 2016;37(25):1967-76.
19. Hilvo M, Salonurmi T, Havulinna AS, Kauhanen D, Pedersen ER, Tell GS, et al. Ceramide stearic to palmitic acid ratio predicts incident diabetes. Diabetologia. 2018;61(6):1424-34.

20. Leiherer A, Mündlein A, Laaksonen R, Lääperi M, Jylhä A, Fraunberger P, et al. Comparison of recent ceramide-based coronary risk prediction scores in cardiovascular disease patients. European Journal of Preventive Cardiology. 2021;29(6):947-56.
21. Hilvo M, Jylhä A, Lääperi M, Jousilahti P, Laaksonen R. Absolute and relative risk prediction in cardiovascular primary prevention with a modified SCORE chart incorporating ceramide-phospholipid risk score and diabetes mellitus. European Heart Journal Open. 2021;1(3).
22. Hilvo M, Vasile VC, Donato LJ, Hurme R, Laaksonen R. Ceramides and Ceramide Scores: Clinical Applications for Cardiometabolic Risk Stratification. Front Endocrinol (Lausanne). 2020;11:570628.
23. Katajamäki TT, Koivula M-K, Hilvo M, Lääperi MTA, Salminen MJ, Viljanen AM, et al. Ceramides and Phosphatidylcholines Associate with Cardiovascular Diseases in the Elderly. Clinical Chemistry. 2022;68(12):1502-8.
24. Nicholls M. Plasma ceramides and cardiac risk. European Heart Journal. 2017;38(18):1359-60.
25. Vasile VC, Jaffe AS. An enhanced ceramide-based approach for primary prevention of atherosclerotic events. European Heart Journal Open. 2021;1(3).
26. Fiuza-Luces C, Garatachea N, Berger NA, Lucia A. Exercise is the Real Polypill. Physiology. 2013;28(5):330-58.
27. Katzmarzyk PT. Cost-effectiveness of exercise is medicine. Curr Sports Med Rep. 2011;10(4):217-23.
28. Bull FC, Al-Ansari SS, Biddle S, Borodulin K, Buman MP, Cardon G, et al. World Health Organization 2020 guidelines on physical activity and sedentary behaviour. Br J Sports Med. 2020;54(24):1451-62.
29. Sarzynski MA, Rice TK, Després JP, Pérusse L, Tremblay A, Stanforth PR, et al. The HERITAGE Family Study: A Review of the Effects of Exercise Training on Cardiometabolic Health, with Insights into Molecular Transducers. Med Sci Sports Exerc. 2022;54(5s):S1-s43.
30. Fiuza-Luces C, Santos-Lozano A, Joyner M, Carrera-Bastos P, Picazo O, Zugaza JL, et al. Exercise benefits in cardiovascular disease: beyond attenuation of traditional risk factors. Nat Rev Cardiol. 2018;15(12):731-43.
31. Newsom SA, Schenk S, Li M, Everett AC, Horowitz JF. High fatty acid availability after exercise alters the regulation of muscle lipid metabolism. Metabolism. 2011;60(6):852-9.
32. Tan-Chen S, Guitton J, Bourron O, Le Stunff H, Hajduch E. Sphingolipid Metabolism and Signaling in Skeletal Muscle: From Physiology to Physiopathology. Frontiers in Endocrinology. 2020;11(491).
33. Carrard J, Guerini C, Appenzeller-Herzog C, Infanger D, Königstein K, Streese L, et al. The metabolic signature of cardiorespiratory fitness: a protocol for a systematic review and meta-analysis. BMJ Open Sport Exerc Med. 2021;7(1):e001008.
34. Ross R, Blair SN, Arena R, Church TS, Després JP, Franklin BA, et al. Importance of Assessing Cardiorespiratory Fitness in Clinical Practice: A Case for Fitness as a Clinical Vital Sign: A Scientific Statement From the American Heart Association. Circulation. 2016;134(24):e653-e99.
35. Kasumov T, Solomon TPJ, Hwang C, Huang H, Haus JM, Zhang R, et al. Improved insulin sensitivity after exercise training is linked to reduced plasma C14:0 ceramide in obesity and type 2 diabetes. Obesity. 2015;23(7):1414-21.
36. Dun Y, Smith JR, Liu S, Olson TP. High-Intensity Interval Training in Cardiac Rehabilitation. Clinics in geriatric medicine. 2019;35(4):469-87.
37. Wewege MA, Ahn D, Yu J, Liou K, Keech A. High-Intensity Interval Training for Patients With Cardiovascular Disease-Is It Safe? A Systematic Review. J Am Heart Assoc. 2018;7(21):e009305.

38. Villelabeitia-Jaureguizar K, Vicente-Campos D, Senen AB, Jiménez VH, Garrido-Lestache MEB, Chicharro JL. Effects of high-intensity interval versus continuous exercise training on post-exercise heart rate recovery in coronary heart-disease patients. Int J Cardiol. 2017;244:17-23.
39. Taylor JL, Holland DJ, Spathis JG, Beetham KS, Wisløff U, Keating SE, et al. Guidelines for the delivery and monitoring of high intensity interval training in clinical populations. Prog Cardiovasc Dis. 2019;62(2):140-6.
40. Weston KS, Wisløff U, Coombes JS. High-intensity interval training in patients with lifestyle-induced cardiometabolic disease: a systematic review and meta-analysis. Br J Sports Med. 2014;48(16):1227-34.
41. Bird SR, Hawley JA. Update on the effects of physical activity on insulin sensitivity in humans. BMJ Open Sport & Exercise Medicine. 2017;2(1):e000143.
42. Campbell WW, Kraus WE, Powell KE, Haskell WL, Janz KF, Jakicic JM, et al. High-Intensity Interval Training for Cardiometabolic Disease Prevention. Med Sci Sports Exerc. 2019;51(6):1220-6.
43. Mendes R, Sousa N, Themudo-Barata JL, Reis VM. High-Intensity Interval Training Versus Moderate-Intensity Continuous Training in Middle-Aged and Older Patients with Type 2 Diabetes: A Randomized Controlled Crossover Trial of the Acute Effects of Treadmill Walking on Glycemic Control. Int J Environ Res Public Health. 2019;16(21).
44. Eddolls WTB, McNarry MA, Stratton G, Winn CON, Mackintosh KA. High-Intensity Interval Training Interventions in Children and Adolescents: A Systematic Review. Sports Med. 2017;47(11):2363-74.
45. Wang M, Wang C, Han RH, Han X. Novel advances in shotgun lipidomics for biology and medicine. Prog Lipid Res. 2016;61:83-108.
46. Yang K, Han X. Lipidomics: Techniques, Applications, and Outcomes Related to Biomedical Sciences. Trends in Biochemical Sciences. 2016;41(11):954-69.
47. Medina J BR, Teav T, Gao L, Ji S, Carrard J, et al. ‘Omic-scale quantitative HILIC-MS/MS approach for circulatory lipid phenotyping in clinical research. ChemRxiv. Cambridge: Cambridge Open Engage; 2022; This content is a preprint and has not been peer-reviewed.
48. Checa A, Khademi M, Sar DG, Haeggström JZ, Lundberg JO, Piehl F, et al. Hexosylceramides as intrathecal markers of worsening disability in multiple sclerosis. Mult Scler. 2015;21(10):1271-9.
49. Efird J. Blocked randomization with randomly selected block sizes. Int J Environ Res Public Health. 2011;8(1):15-20.
50. Wisløff U, Støylen A, Loennechen JP, Bruvold M, Rognmo Ø, Haram PM, et al. Superior Cardiovascular Effect of Aerobic Interval Training Versus Moderate Continuous Training in Heart Failure Patients. Circulation. 2007;115(24):3086-94.
51. Williams CJ, Gurd BJ, Bonafiglia JT, Voisin S, Li Z, Harvey N, et al. A Multi-Center Comparison of O2peak Trainability Between Interval Training and Moderate Intensity Continuous Training. Frontiers in Physiology. 2019;10.
52. Karlsen T, Aamot IL, Haykowsky M, Rognmo Ø. High Intensity Interval Training for Maximizing Health Outcomes. Prog Cardiovasc Dis. 2017;60(1):67-77.
53. Streese L, Khan AW, Deiseroth A, Hussain S, Suades R, Tiaden A, et al. High-intensity interval training modulates retinal microvascular phenotype and DNA methylation of p66Shc gene: a randomized controlled trial (EXAMIN AGE). European Heart Journal. 2019;41(15):1514-9.
54. Streese L, Gander J, Carrard J, Hauser C, Hinrichs T, Schmidt-Trucksäss A, et al. Hypertension and retinal microvascular dysfunction (HyperVasc): protocol of a randomised controlled exercise trial in patients with hypertension. BMJ Open. 2022;12(6):e058997.

55. Hecksteden A, Faude O, Meyer T, Donath L. How to Construct, Conduct and Analyze an Exercise Training Study? Front Physiol. 2018;9:1007.
56. Piepoli MF, Hoes AW, Agewall S, Albus C, Brotons C, Catapano AL, et al. 2016 European Guidelines on cardiovascular disease prevention in clinical practice: The Sixth Joint Task Force of the European Society of Cardiology and Other Societies on Cardiovascular Disease Prevention in Clinical Practice (constituted by representatives of 10 societies and by invited experts)Developed with the special contribution of the European Association for Cardiovascular Prevention & Rehabilitation (EACPR). European Heart Journal. 2016;37(29):2315-81.
57. Armstrong T, Bull F. Development of the World Health Organization Global Physical Activity Questionnaire (GPAQ). Journal of Public Health. 2006;14(2):66-70.
58. Wanner M, Hartmann C, Pestoni G, Martin BW, Siegrist M, Martin-Diener E. Validation of the Global Physical Activity Questionnaire for self-administration in a European context. BMJ Open Sport & Exercise Medicine. 2017;3(1):e000206.
59. Marra M, Sammarco R, De Lorenzo A, Iellamo F, Siervo M, Pietrobelli A, et al. Assessment of Body Composition in Health and Disease Using Bioelectrical Impedance Analysis (BIA) and Dual Energy X-Ray Absorptiometry (DXA): A Critical Overview. Contrast Media Mol Imaging. 2019;2019:3548284.
60. Shepherd JA, Ng BK, Sommer MJ, Heymsfield SB. Body composition by DXA. Bone. 2017;104:101-5.
61. Gayoso-Diz P, Otero-Gonzalez A, Rodriguez-Alvarez MX, Gude F, Cadarso-Suarez C, García F, et al. Insulin resistance index (HOMA-IR) levels in a general adult population: Curves percentile by gender and age. The EPIRCE study. Diabetes Research and Clinical Practice. 2011;94(1):146-55.
62. Mifflin MD, St Jeor ST, Hill LA, Scott BJ, Daugherty SA, Koh YO. A new predictive equation for resting energy expenditure in healthy individuals. Am J Clin Nutr. 1990;51(2):241-7.
63. Hall KD, Sacks G, Chandramohan D, Chow CC, Wang YC, Gortmaker SL, et al. Quantification of the effect of energy imbalance on bodyweight. Lancet. 2011;378(9793):826-37.
64. Hall KD, Chow CC. Estimating changes in free-living energy intake and its confidence interval. Am J Clin Nutr. 2011;94(1):66-74.
65. Hanssen H, Streese L, Vilser W. Retinal vessel diameters and function in cardiovascular risk and disease. Progress in Retinal and Eye Research. 2022:101095.
66. Hubbard LD, Brothers RJ, King WN, Clegg LX, Klein R, Cooper LS, et al. Methods for evaluation of retinal microvascular abnormalities associated with hypertension/sclerosis in the Atherosclerosis Risk in Communities Study. Ophthalmology. 1999;106(12):2269-80.
67. Wong TY, Knudtson MD, Klein R, Klein BE, Meuer SM, Hubbard LD. Computer-assisted measurement of retinal vessel diameters in the Beaver Dam Eye Study: methodology, correlation between eyes, and effect of refractive errors. Ophthalmology. 2004;111(6):1183-90.
68. Vlachopoulos C, Xaplanteris P, Aboyans V, Brodmann M, Cifkova R, Cosentino F, et al. The role of vascular biomarkers for primary and secondary prevention. A position paper from the European Society of Cardiology Working Group on peripheral circulation: Endorsed by the Association for Research into Arterial Structure and Physiology (ARTERY) Society. Atherosclerosis. 2015;241(2):507-32.
69. Thijssen DHJ, Bruno RM, van Mil A, Holder SM, Faita F, Greyling A, et al. Expert consensus and evidence-based recommendations for the assessment of flow-mediated dilation in humans. European heart journal. 2019;40(30):2534-47.
70. Holder SM, Bruno RM, Shkredova DA, Dawson EA, Jones H, Hopkins ND, et al. Reference Intervals for Brachial Artery Flow-Mediated Dilation and the Relation With Cardiovascular Risk Factors. Hypertension. 2021;77(5):1469-80.
71. Yubero-Serrano EM, Fernandez-Gandara C, Garcia-Rios A, Rangel-Zuniga OA, Gutierrez-Mariscal FM, Torres-Pena JD, et al. Mediterranean diet and endothelial function in

patients with coronary heart disease: An analysis of the CORDIOPREV randomized controlled trial. PLoS Med. 2020;17(9):e1003282.
72. Xue YT, Tan QW, Li P, Mou SF, Liu SJ, Bao Y, et al. Investigating the role of acute mental stress on endothelial dysfunction: a systematic review and meta-analysis. Clin Res Cardiol. 2015;104(4):310-9.
73. Dawson EA, Sheikhsaraf B, Boidin M, Erskine RM, Thijssen DHJ. Intra-individual differences in the effect of endurance versus resistance training on vascular function: A cross-over study. Scand J Med Sci Sports. 2021;31(8):1683-92.
74. Charakida M, de Groot E, Loukogeorgakis SP, Khan T, Luscher T, Kastelein JJ, et al. Variability and reproducibility of flow-mediated dilatation in a multicentre clinical trial. European heart journal. 2013;34(45):3501-7.
75. Tomiyama H, Kohro T, Higashi Y, Takase B, Suzuki T, Ishizu T, et al. Reliability of measurement of endothelial function across multiple institutions and establishment of reference values in Japanese. Atherosclerosis. 2015;242(2):433-42.
76. Hauser R, Nielsen AB, Skaarup KG, Lassen MCH, Duus LS, Johansen ND, et al. Left atrial strain predicts incident atrial fibrillation in the general population: the Copenhagen City Heart Study. Eur Heart J Cardiovasc Imaging. 2021;23(1):52-60.
77. Lavie CJ, Pandey A, Lau DH, Alpert MA, Sanders P. Obesity and Atrial Fibrillation Prevalence, Pathogenesis, and Prognosis: Effects of Weight Loss and Exercise. J Am Coll Cardiol. 2017;70(16):2022-35.
78. Li CY, Zhang JR, Hu WN, Li SN. Atrial fibrosis underlying atrial fibrillation (Review). Int J Mol Med. 2021;47(3).
79. Elliott AD, Ariyaratnam J, Howden EJ, La Gerche A, Sanders P. Influence of exercise training on the left atrium: implications for atrial fibrillation, heart failure, and stroke. Am J Physiol Heart Circ Physiol. 2023;325(4):H822-h36.
80. Mitchell C, Rahko PS, Blauwet LA, Canaday B, Finstuen JA, Foster MC, et al. Guidelines for Performing a Comprehensive Transthoracic Echocardiographic Examination in Adults: Recommendations from the American Society of Echocardiography. J Am Soc Echocardiogr. 2019;32(1):1-64.
81. Badano LP, Kolias TJ, Muraru D, Abraham TP, Aurigemma G, Edvardsen T, et al. Standardization of left atrial, right ventricular, and right atrial deformation imaging using two-dimensional speckle tracking echocardiography: a consensus document of the EACVI/ASE/Industry Task Force to standardize deformation imaging. Eur Heart J Cardiovasc Imaging. 2018;19(6):591-600.
82. Streese L, Deiseroth A, Schäfer J, Schmidt-Trucksäss A, Hanssen H. Exercise, Arterial Crosstalk-Modulation, and Inflammation in an Aging Population: The ExAMIN AGE Study. Front Physiol. 2018;9:116.
83. Liu J, Liu X, Luo Y, Huang F, Xie Y, Zheng S, et al. Sphingolipids: drivers of cardiac fibrosis and atrial fibrillation. J Mol Med (Berl). 2023.
84. Carrard J, Gallart-Ayala H, Infanger D, Teav T, Wagner J, Knaier R, et al. Metabolic View on Human Healthspan: A Lipidome-Wide Association Study. Metabolites. 2021;11(5):287.
85. Vickers AJ, Altman DG. Analysing controlled trials with baseline and follow up measurements. BMJ. 2001;323(7321):1123-4.
86. Textor J, van der Zander B, Gilthorpe MS, Liśkiewicz M, Ellison GT. Robust causal inference using directed acyclic graphs: the R package ‘dagitty’. International Journal of Epidemiology. 2017;45(6):1887-94.
87. Cinelli C, Forney A, Pearl J. A crash course in good and bad controls. Available at SSRN 3689437. 2020.
88. Hochsmann C, Rossmeissl A, Baumann S, Infanger D, Schmidt-Trucksass A. Oxygen uptake during mini trampoline exercise in normal-weight, endurance-trained adults and in

overweight-obese, inactive adults: A proof-of-concept study. European journal of sport science. 2018;18(5):753-61.
89. Schulz KF, Altman DG, Moher D. CONSORT 2010 Statement: updated guidelines for reporting parallel group randomised trials. BMJ. 2010;340:c332.
