# Supplementary material for: Effect of an eight-week high-intensity interval training programme on circulating sphingolipid levels in middle-aged adults at elevated cardiometabolic risk (SphingoFIT)—Protocol for a randomised controlled exercise trial
Source: PLoS One. 2024 May 8;19(5):e0302477. doi: 10.1371/journal.pone.0302477 (PMC11078397; doi:10.1371/journal.pone.0302477)
Supplement: S2 File — (PDF) [file pone.0302477.s003.pdf]

CRSK-3_220309
Carrard, Justin

Reducing Circulating Sphingolipid Levels to Optimise Cardiometabolic Health - The SphingoFIT Randomised Controlled Trial

Data management plan (DMP)

1 Data collection and documentation

1.1 What data will you collect, observe, generate or reuse?

The following data will be collected at baseline and following the intervention for all 98 participants to be included:
- participants' demographic data (sex, age, height, weight, body mass index),
- habitual physical activity,
- vital signs (temperature, respiratory rate, heart rate, arterial blood pressure, oxygen saturation),
- resting ECG,
- cardiopulmonary exercise testing (including an exercise ECG),
- compliance with the standardised feeding programme,
- body composition,
- retinal vessel diameter assessment,
- endothelial function of the brachial arteries,
- strain assessment of the heart cavities,
- attendance of the supervised training,
- compliance with the supervised training intensity,
- plasma sphingolipid concentration,
- serum concentration of total cholesterol, low-density lipoprotein cholesterol, high-density lipoprotein cholesterol, triglycerides, HbA1c, glucose, insulin and insulin resistance,
No existing data will be reused.

1.2 How will the data be collected, observed or generated?

The data will be collected as follows:
- sex and age will be asked orally,
- height and weight will be measured to the nearest 0.5 cm and 0.1 kg, respectively,
- body mass index will be calculated as kg/m2,
- habitual physical activity will be assessed using the Global Physical Activity Questionnaire,
- vital signs (temperature, respiratory rate, heart rate, arterial blood pressure, oxygen saturation) will be measured clinically after a rest phase of 5 min in the supine position,
- resting ECG and cardiopulmonary exercise testing will be assessed clinically (Ergoselect 200, Ergoline, Bitz, Germany; MetaMax 3B; Cortex Biophysik GmbH, Leipzig, Germany),
- to monitor diet adherence, participants will be instructed to return all non-consumed foods from the pre-packaged meals to the lab and take photos of additionally consumed foods for later analysis. Compliance will be expressed in percentages comparing what was effectively consumed vs. what should have been consumed,
- body composition will be assessed using bioelectrical impedance analysis (Inbody Co. Ltd., Seoul, South Korea) and Dual-energy X-ray absorptiometry (GE Lunar Inc., Madison, WI; software version 13.10),
- retinal vessel diameter will be assessed using non-invasive analysis of static retinal vessel diameters (450FF; Carl Zeiss, Jena, Germany and Vesselmap 2; IMEDOS Systems, Jena, Germany),
- endothelial function of the brachial arteries will be assessed using non-invasive flow-mediated dilatation (EF, Unex Corporation, Nagoya, Japan),
- strain assessment of the heart cavities will be assessed using transthoracic echocardiography (Philips Epiq 7 Ultrasound System, Koninklijke Philips N.V., Amsterdam; S5-1, Philips, Koninklijke Philips N.V., Amsterdam; TOMTEC Imaging Systems GmbH, Unterschleissheim, Germany),
- attendance of the supervised training will be observed,
- compliance with the individualised training intensity will be assessed using Garmin HRM-Dual heart rate sensors combined with Garmin Forerunner 45S watches (Garmin, USA)

- plasma sphingolipid concentration will be quantified using reversed-phase liquid chromatography coupled to tandem mass spectrometry (Thermo Scientific, San Jose, CA, United States),
- total cholesterol, low-density lipoprotein cholesterol, high-density lipoprotein cholesterol, triglycerides, HbA1c, glucose, insulin and insulin resistance (using the Homeostatic Model Assessment for Insulin Resistance) will be assessed using standardised analytics chemistry processes.
- Case Report Forms will be updated at each participant's visit, and collected data will be saved after each participant's visit on the protected server of the Department (see below).

1.3 What documentation and metadata will you provide with the data?

All raw data and metadata will be made freely accessible on a depository (see below) when the respective scientific output is published at the latest.
README files will complement the metadata to explicit each variable name and unit.
Each file will be named and identified by a persistent identifier.
The name of the person who collected or contributed to the data, the collection date, and the conditions for accessing the data will be openly disclosed.
All methodological information necessary to replicate the study will be contained in the study protocol, which will be published in an Open Access Journal.

2 Ethics, legal and security issues

2.1 How will ethical issues be addressed and handled?

The Ethics Committee of Northwest and Central Switzerland approved this protocol (EKNZ 2023–01345).

A Case Report Form will be created for each participant in which the participant's data is only stored in encrypted form. No conclusions about personal data such as name, date of birth or telephone number can be drawn from this. Indeed, each enrolled participant will be pseudonymised by a unique subject identifier (participant ID).

All involved parties will keep the participant’s data strictly confidential. Data will be entered in protected files on the server of the Department of Sport, Exercise and Health of the University of Basel. Access to these files will be restricted to study group members who agreed on confidentiality.

In the SphingoFIT study, only coded non-genetic data will be used. Biological material in this study will not be identified by participant name but by a unique participant number. Biological material will be appropriately stored in a restricted area only accessible to authorised personnel.

Participants are informed about how their data will be obtained, processed, preserved and shared and will provide written consent.

2.2 How will data access and security be managed?

All data will be pseudonymised with a unique subject identifier (participant ID) and stored in protected files on the Department of Sport, Exercise and Health server of the University of Basel. Pseudonymised data will be only accessible to authorised personnel who require the data to fulfil their duties within the scope of the study. Authorisation to access the data will be delivered in a two-step process in which both the Principal Investigator and the Head of the Department will have to grant authorisation access.

The participant identification list will only be stored on the server of the Department of Sport, Exercise and Health of the University of Basel. Access to it will be restricted to the Principal Investigator and selected study personnel if contact with participants is required.

2.3 How will you handle copyright and Intellectual Property Rights issues?

The Principal Investigator will be the owner of the data.
Data will be made publicly available under the Creative Commons Attribution 4.0 International licence when the respective scientific output is published at the latest.
No third-party data will be reused.

3 Data storage and preservation

3.1 How will your data be stored and backed-up during the research?

All study data will be archived locally in a password-protected file on a local server for ten years after study termination or premature termination of the study at the Department of Sport, Exercise and Health of the University of Basel. Biological material collected during the SphingoFIT study will be stored at the Department of Sport, Exercise and Health for ten years after study termination. Biological material will be eliminated after these ten years, and the destruction of this material will be documented using a confirmation sheet, which will be stored locally on a computer at the Department of Sport, Exercise and Health of the University of Basel.

3.2 What is your data preservation plan?

All pseudonymised data will be made publicly available on a repository (see below) under the Creative Commons Attribution 4.0 International licence when the respective scientific output is published at the latest. The study protocol, containing all methodological information necessary to replicate the study, will be published in an Open Access Journal.

Biological material will be eliminated after these ten years, and the destruction of this material will be documented using a confirmation sheet, which will be stored locally on a computer at the Department of Sport, Exercise and Health of the University of Basel.

4 Data sharing and reuse

4.1 How and where will the data be shared?

To analyse the circulating sphingolipids using RPLC-MS/MS, plasma probes, identified by the participant's ID, will be sent to the Metabolomics Platform of the University of Lausanne.

Dr Julijana Ivanisevic, head of the Metabolomics Platform, will receive the probes. She will guarantee confidentiality during the whole analysis duration when handling the investigations and the resulting data.

4.2 Are there any necessary limitations to protect sensitive data?

Anonymised data will be made openly accessible in a repository (see below) when the respective scientific output is published at the latest.

4.3 All digital repositories I will choose are conform to the FAIR Data Principles.

Yes

4.4 I will choose digital repositories maintained by a non-profit organisation.

Yes
