# Supplementary material for: Effect of an eight-week high-intensity interval training programme on circulating sphingolipid levels in middle-aged adults at elevated cardiometabolic risk (SphingoFIT)—Protocol for a randomised controlled exercise trial
Source: PLoS One. 2024 May 8;19(5):e0302477. doi: 10.1371/journal.pone.0302477 (PMC11078397; doi:10.1371/journal.pone.0302477)
Supplement: Peer review history

# Editor report: Decision Letter 0

**DOI:** 10.1371/journal.pone.0302477.r001

**Authors:** Emma Campbell

5 Apr 2024

Effect of an eight-week high-intensity interval training programme on circulating sphingolipid levels in middle-aged adults at elevated cardiometabolic risk (SphingoFIT) – Protocol for a randomised controlled exercise trial

PONE-D-24-08816

Dear Dr. Carrard,

As a Peer-Reviewed Funded Protocol, your submission is eligible for expedited review by in-house editors. Based on our evaluation, we are satisfied that your manuscript meets our publication criteria for Study Protocols, and is therefore considered to be suitable for publication subject to final journal requirements.

We’re pleased to inform you that your manuscript has been judged scientifically suitable for publication and will be formally accepted for publication once it meets all outstanding technical requirements.

Within one week, you’ll receive an e-mail detailing the required amendments. When these have been addressed, you’ll receive a formal acceptance letter and your manuscript will be scheduled for publication.

An invoice will be generated when your article is formally accepted. Please note, if your institution has a publishing partnership with PLOS and your article meets the relevant criteria, all or part of your publication costs will be covered. Please make sure your user information is up-to-date by logging into Editorial Manager at Editorial Manager® and clicking the ‘Update My Information' link at the top of the page. If you have any questions relating to publication charges, please contact our Author Billing department directly at authorbilling@plos.org.

If your institution or institutions have a press office, please notify them about your upcoming paper to help maximize its impact. If they’ll be preparing press materials, please inform our press team as soon as possible -- no later than 48 hours after receiving the formal acceptance. Your manuscript will remain under strict press embargo until 2 pm Eastern Time on the date of publication. For more information, please contact onepress@plos.org.

Kind regards,

Emma Campbell, Ph.D

Staff Editor

PLOS ONE

Journal requirements:

1. Thank you for stating in your Funding Statement:

“This study is supported by the Swiss National Research Foundation (awarded to JD, ID CRSK-3_220309, https://www.snf.ch/en/8EWqXT6CZ7wuAJUq/funding/programmes/spark), by the Research Fund of the University of Basel for Excellent Junior Researchers (awarded to JC, ID U.360.0002, https://www.unibas.ch/en/Research/Individual-Funding/Research-Fund.html) and by the Swiss Life Jubilee Foundation (awarded to JC, no ID, https://www.swisslife.ch/de/ueber-uns/engagement/stiftungen/jubilaeumsstiftung.html). The funders had no role in study design, data collection and analysis, publication decisions, or manuscript preparation.”

Please provide an amended statement that declares *all* the funding or sources of support (whether external or internal to your organization) received during this study, as detailed online in our guide for authors at http://journals.plos.org/plosone/s/submit-now.  Please also include the statement “There was no additional external funding received for this study.” in your updated Funding Statement.

Please respond by return e-mail so that we can amend your financial disclosure and competing interests on your behalf.

2. Your ethics statement should only appear in the Methods section of your manuscript. If your ethics statement is written in any section besides the Methods, please delete it from any other section.
